# USP10 mitigates Ang II–induced atrial remodeling and atrial fibrillation susceptibility by deubiquitinating NDUFS1

**DOI:** 10.1172/jci.insight.195761

**Published:** 2026-08-24

**Authors:** Wanrong Fu, Xiao-Xu Tian, Jianghua Zhou, Yu-Xu Huang, Huan Li, Zhenya Wang, Tong-You Wade Wei, Li Li, Guo-Jun Zhao

**Affiliations:** 1 Department of Cardiology, The First Affiliated Hospital of Zhengzhou University, Zhengzhou, China.; 2 Department of Cardiology, The First Affiliated Hospital of Wenzhou Medical University, Wenzhou, China.; 3 Department of Medicine, JingGangShan University, Ji’an, China.; 4 Division of Cardiology, Department of Medicine, UCSD, La Jolla, California, USA.; 5 Department of Cardiovascular Surgery, The First Affiliated Hospital of Zhengzhou University, Zhengzhou, China.

**Keywords:** Cardiology, Vascular biology, Arrhythmias, Cardiovascular disease, Ubiquitin-proteosome system

## Abstract

Atrial fibrillation (AF) contributes to cardiovascular morbidity and mortality. Ubiquitin-specific peptidase 10 (USP10) plays a crucial role in numerous cellular processes; however, its particular role in AF remains largely unexplored. In the present study, USP10 expression was assessed in human atrial samples and angiotensin II–treated (Ang II–treated) mouse atrial tissues. An Ang II–induced AF mouse model was employed to investigate the effects of USP10 on atrial remodeling and AF susceptibility. Calcium imaging and patch clamp techniques were used to evaluate USP10’s influence on calcium handling and triggered activity. Additionally, RNA sequencing, coimmunoprecipitation, and ubiquitination assays were performed to explore the regulatory interactions between USP10 and NADH:ubiquinone oxidoreductase subunit S1 (NDUFS1). Our findings demonstrate that USP10 is downregulated in atrial tissues from mouse models and patients with AF. USP10 overexpression counteracts Ang II–induced atrial remodeling and reduces AF susceptibility. Furthermore, USP10 contributes to the restoration of mitochondrial function in AF. Mechanistically, USP10 deubiquitinates NDUFS1 at lysine 621, stabilizing NDUFS1 protein levels and mitigating Ang II–induced mitochondrial dysfunction. This study uncovers a critical mechanistic link between USP10 and NDUFS1. Our findings suggest that upregulating USP10 or targeting NDUFS1 degradation could provide an alternative therapeutic strategy to mitigate AF progression and associated cardiovascular risk.

## Abstract 2

USP10 de-ubiquitinates NDUFS1 in AF

## Graphical abstract

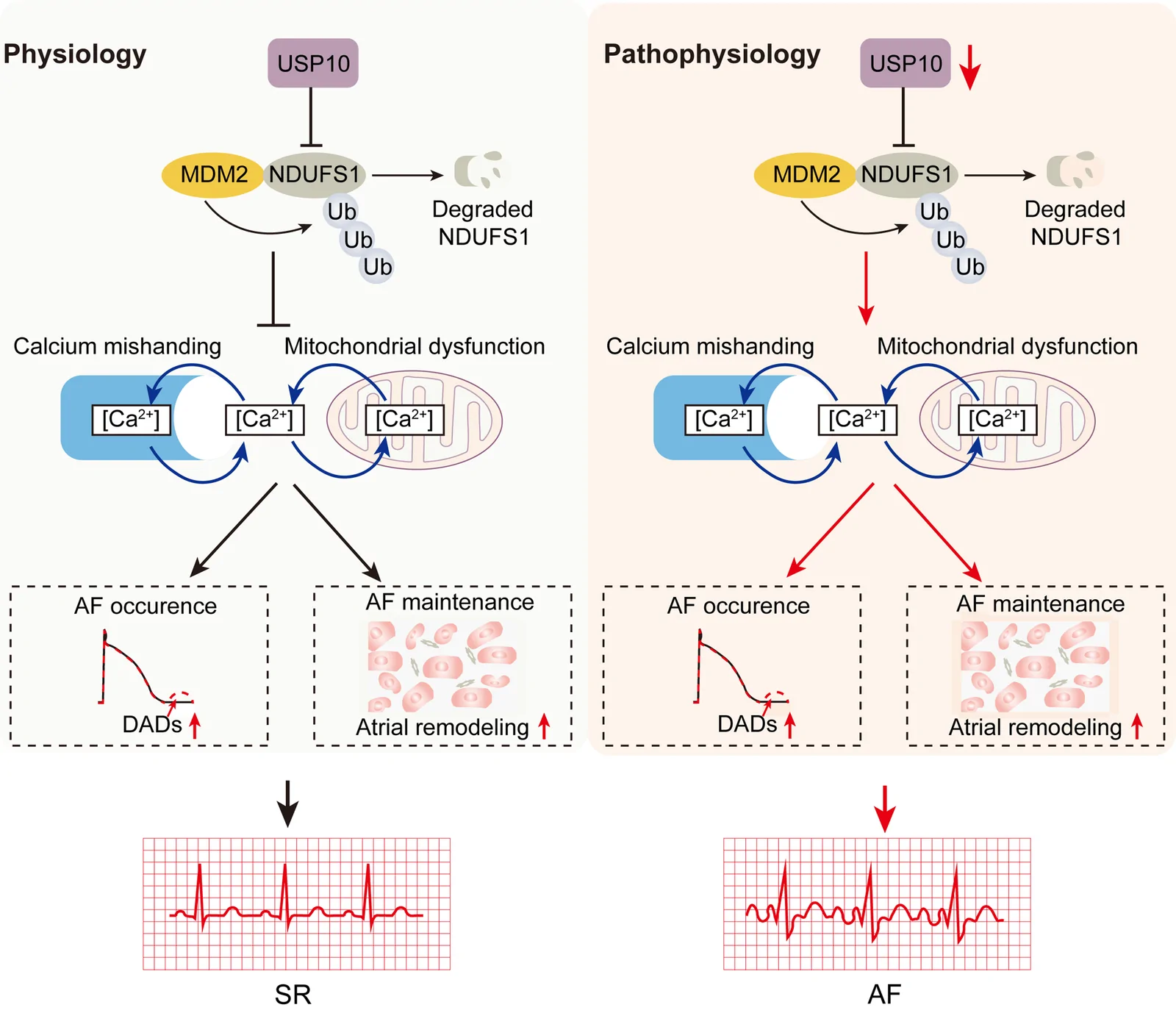

## Introduction

Atrial fibrillation (AF) is the most prevalent arrhythmia, with its global incidence steadily increasing (1). In addition to markedly impairing patients’ quality of life, AF plays a critical role in cardiovascular morbidity and mortality (2, 3). Although substantial progress has been made in AF treatments over the past few decades, safe and effective therapies remain elusive. Furthermore, the molecular mechanisms underlying AF pathogenesis are not yet fully understood, highlighting the need for a deeper understanding to develop alternative therapeutic strategies.

The pathophysiology of AF is intricate, but growing evidence indicates that mitochondrial dysfunction plays a pivotal role in the onset and persistence of the arrhythmia (4). Mitochondria are essential for ATP production, which fuels cardiomyocyte excitation-contraction coupling and a range of intracellular processes (4, 5). When mitochondrial function is compromised, the high ATP demands of frequent atrial depolarizations may not be met, leading to contractile dysfunction and the progression of AF (4, 5). Additionally, mitochondrial dysfunction disrupts Ca^2+^ homeostasis, elevates oxidative stress, and triggers inflammatory responses, all of which contribute to the arrhythmogenic substrate of AF (6–8). Mitochondrial dysfunction also promotes cardiomyocyte hypertrophy, fibroblast activation, and extracellular matrix deposition, further facilitating atrial remodeling (9, 10). Therefore, maintaining mitochondrial function is crucial for effective AF therapy.

Ubiquitination, a vital posttranslational modification, regulates protein degradation, localization, and activity (11). Ubiquitin-specific peptidase 10 (USP10), a member of the deubiquitinase family, plays a critical role in numerous cellular processes (12), including the regulation of mitochondrial function. USP10 has been shown to deubiquitinate and stabilize mitochondrial proteins, thus playing a key role in maintaining mitochondrial homeostasis (13–15). Notably, USP10 has demonstrated protective effects in various cardiovascular diseases, including cardiac hypertrophy (16), diabetic cardiomyopathy (17), and dilated cardiomyopathy (18). However, its role in AF remains unexplored. Given that the renin-angiotensin system is upregulated in cardiovascular diseases and that angiotensin II (Ang II) is a key driver in AF development and persistence (19, 20), we aimed to investigate the function and underlying mechanisms of USP10 in an Ang II–induced AF mouse model.

Here, we demonstrate that USP10 is significantly downregulated in atrial tissues from both mouse models and patients with AF. Moreover, USP10 overexpression prevents Ang II–induced atrial remodeling and reduces AF susceptibility. Mechanistically, USP10 deubiquitinates and stabilizes NADH:ubiquinone oxidoreductase subunit S1 (NDUFS1), thereby mitigating mitochondrial dysfunction. These findings suggest that upregulating USP10 or preventing NDUFS1 degradation may offer promising therapeutic strategies for AF.

## Results

### USP10 is decreased in AF.

**Figure 1:**
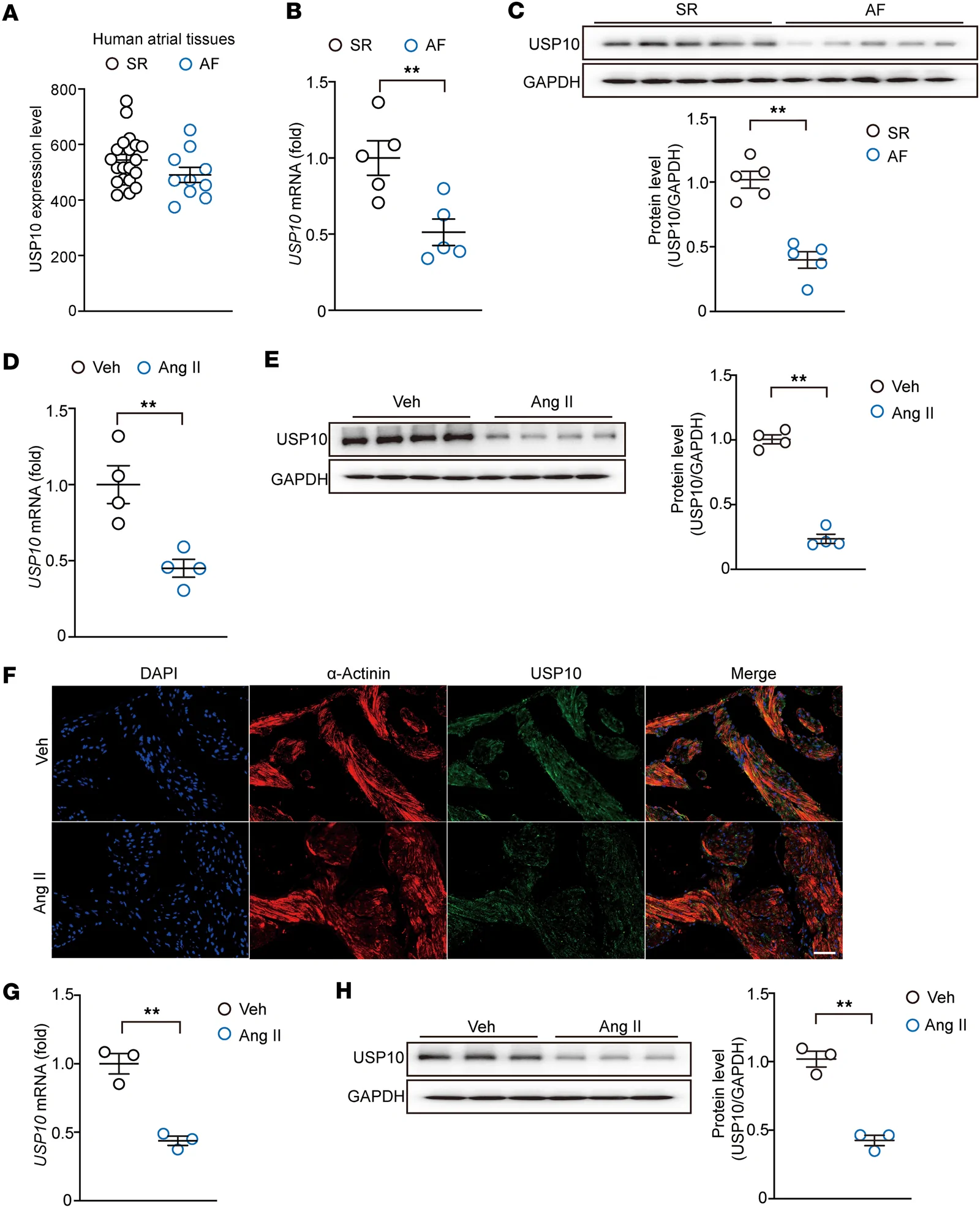
USP10 is decreased in patients with AF and Ang II–treated atrial cardiomyocytes. (**A**)The expression levels of *USP10* in atrial tissues from patients with AF (*n* = 10) and SR controls (*n* = 20). (**B**) qPCR analysis of *USP10* mRNA levels in atrial tissues from patients with AF and SR controls (*n* = 5/group). (**C**) Western blot and quantification of USP10 protein levels in atrial tissues from patients with AF and SR controls (*n* = 5/group). (**D**) qPCR analysis of *Usp10* mRNA levels in atrial tissues from mice treated with Ang II or Veh (*n* = 4/group). (**E**) Representative Western blots and quantification of USP10 in atrial tissues from Ang II– or Veh-treated mice (*n* = 4/group). (**F**) Representative immunostaining images of atrial tissues from Ang II– or Veh-treated mice. Scale bar: 50 μm. (**G**) qPCR analysis of *Usp10* mRNA levels in HL-1 atrial myocytes treated with Ang II or Veh (*n* = 3/group). (**H**) Representative Western blots and quantification of USP10 protein levels in Ang II– or Veh-treated HL-1 atrial myocytes (*n* = 3/group). Data represent mean ± SEM. All statistical comparisons were performed using a 2-tailed Student’s *t* test. **P < 0.01. USP10, ubiquitin-specific peptidase 10; FPKM, fragments per kilobase of exon per million mapped reads; qPCR, quantitative PCR; AF, atrial fibrillation; SR, sinus rhythm; Veh, vehicle; Ang II, angiotensin II.

To investigate the involvement of USP10 in AF, we analyzed an NCBI Gene Expression Omnibus (GEO) RNA-seq dataset (GSE2240) derived from atrial tissues of patients with AF and sinus rhythm (SR) controls (21). Our analysis revealed that USP10 levels were lower in atrial tissues from patients with AF compared with SR controls (Figure 1A). To validate this finding, we collected atrial tissues from patients with AF and SR, assessing USP10 mRNA and protein levels. Consistently, both mRNA and protein levels of USP10 were significantly reduced in atrial tissues from AF patients compared with SR controls (Figure 1, B and C). To determine whether this observation is recapitulated in experimental models, we examined whether this reduction is reproduced in an Ang II–induced mouse model of AF. Consistent with findings in human samples, USP10 mRNA and protein levels were markedly reduced in the atrial tissues of Ang II–treated mice compared with controls (Figure 1, D and E). Immunostaining further confirmed that the reduction in USP10 was specific to cardiomyocytes (Figure 1F). Moreover, cultured HL-1 atrial myocytes demonstrated a significant downregulation of USP10 following Ang II treatment (Figure 1, G and H). Collectively, these findings indicate that USP10 is consistently downregulated in atrial tissues from patients with AF and Ang II–treated mouse models, suggesting a potential contributory role of USP10 deficiency in the pathogenesis of AF.

### USP10 ameliorates Ang II–induced atrial remodeling and reduces AF susceptibility.

**Figure 2:**
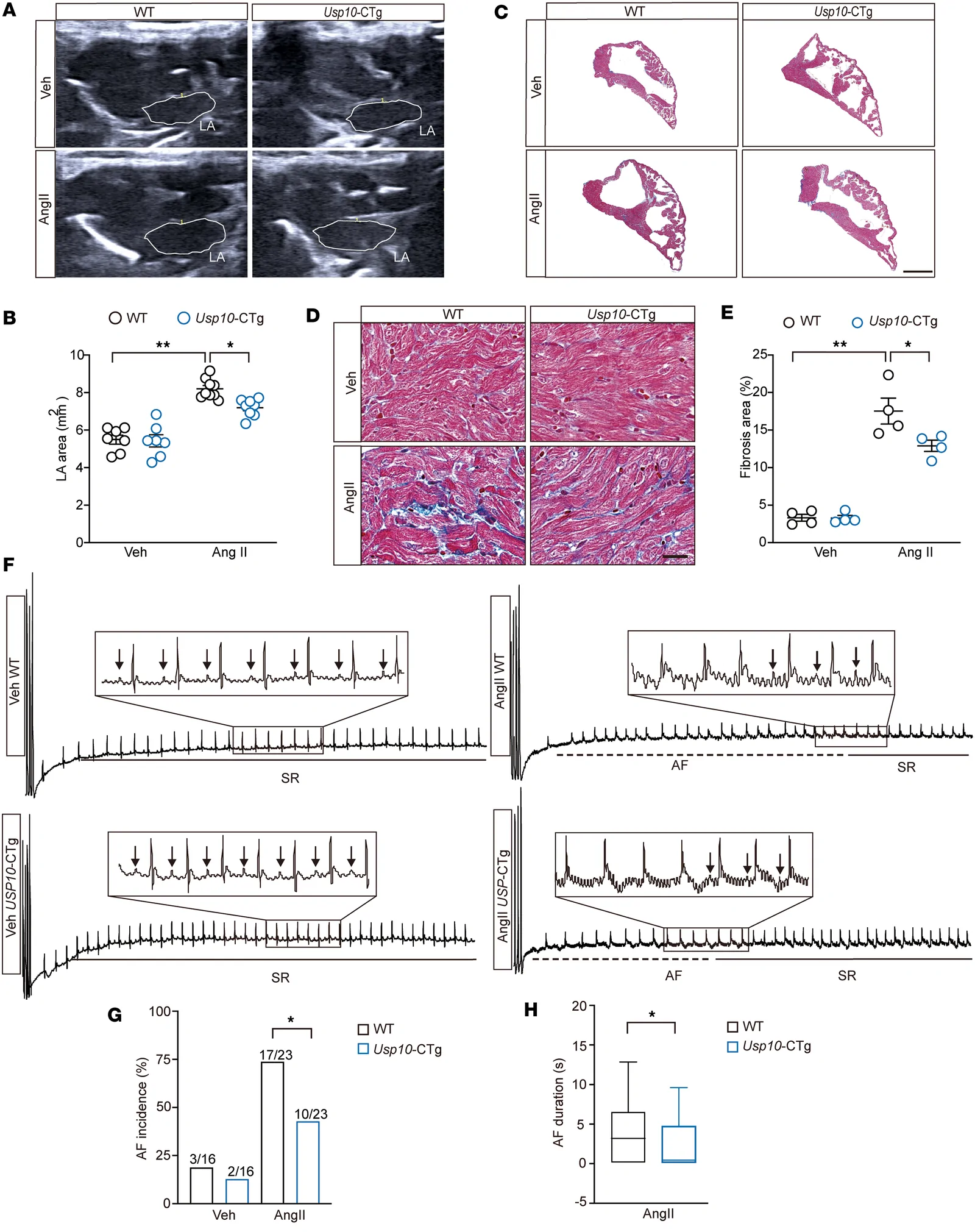
USP10 overexpression ameliorates Ang II–induced atrial remodeling and AF susceptibility. (**A** and **B**) Representative echocardiography images and quantification of left atrial size in WT and *Usp10*-CTg mice subjected to Veh or Ang II treatment (*n* = 7–9/group). (**C**–**E**) Representative images and quantification of Masson’s trichrome staining in left atrial sections from *Usp10*-CTg and WT control mice treated with Veh or Ang II (*n* = 4/group). Scale bars: 1 mm (**C**) and 100 μm (**D**). (**F**) Representative limb lead ECG traces from *Usp10*-CTg and WT control mice in response to Veh or Ang II treatment. (**G**) Fraction of mice with successful AF induction relative to the total number. (**H**) Quantification of AF duration in *Usp10*-CTg and WT control mice treated with Ang II (*n* = 23/group). Data are presented as mean ± SEM and were analyzed using 2-way ANOVA followed by Tukey’s multiple-comparison test (**B** and **E**), χ^2^ test (**G**), and 2-tailed Student’s *t* test (**H**). *P < 0.05, **P < 0.01. USP10, ubiquitin-specific peptidase 10; AF, atrial fibrillation; Ang II, angiotensin II; ECG, electrocardiogram; CTg, cardiomyocyte-specific transgenic.

Given the decreased levels of USP10 in atrial cardiomyocytes, we utilized cardiomyocyte-specific *Usp10*-transgenic (*Usp10*-CTg) mice to investigate the role of USP10 in atrial remodeling and AF susceptibility. As illustrated in Figure 2, A–C, Ang II treatment resulted in a significant increase in left atrial size, while overexpression of *Usp10* notably attenuated this Ang II–induced atrial enlargement. Additionally, we assessed atrial fibrosis, a key marker of atrial remodeling, using Masson’s trichrome staining. This analysis revealed that *Usp10* overexpression effectively suppressed Ang II–induced atrial fibrosis (Figure 2, D and E). To further evaluate the impact of USP10 on AF susceptibility, we conducted transesophageal electrode catheter burst stimulation experiments. Ang II treatment increased both the incidence and duration of AF (Figure 2, F–H). Crucially, overexpression of *Usp10* significantly reduced the incidence rate and duration of Ang II–induced AF (Figure 2, F–H). These results demonstrate that USP10 overexpression mitigates Ang II–induced atrial remodeling and decreases AF susceptibility.

### USP10 overexpression reduces delayed afterdepolarizations and improves calcium handling.

**Figure 3:**
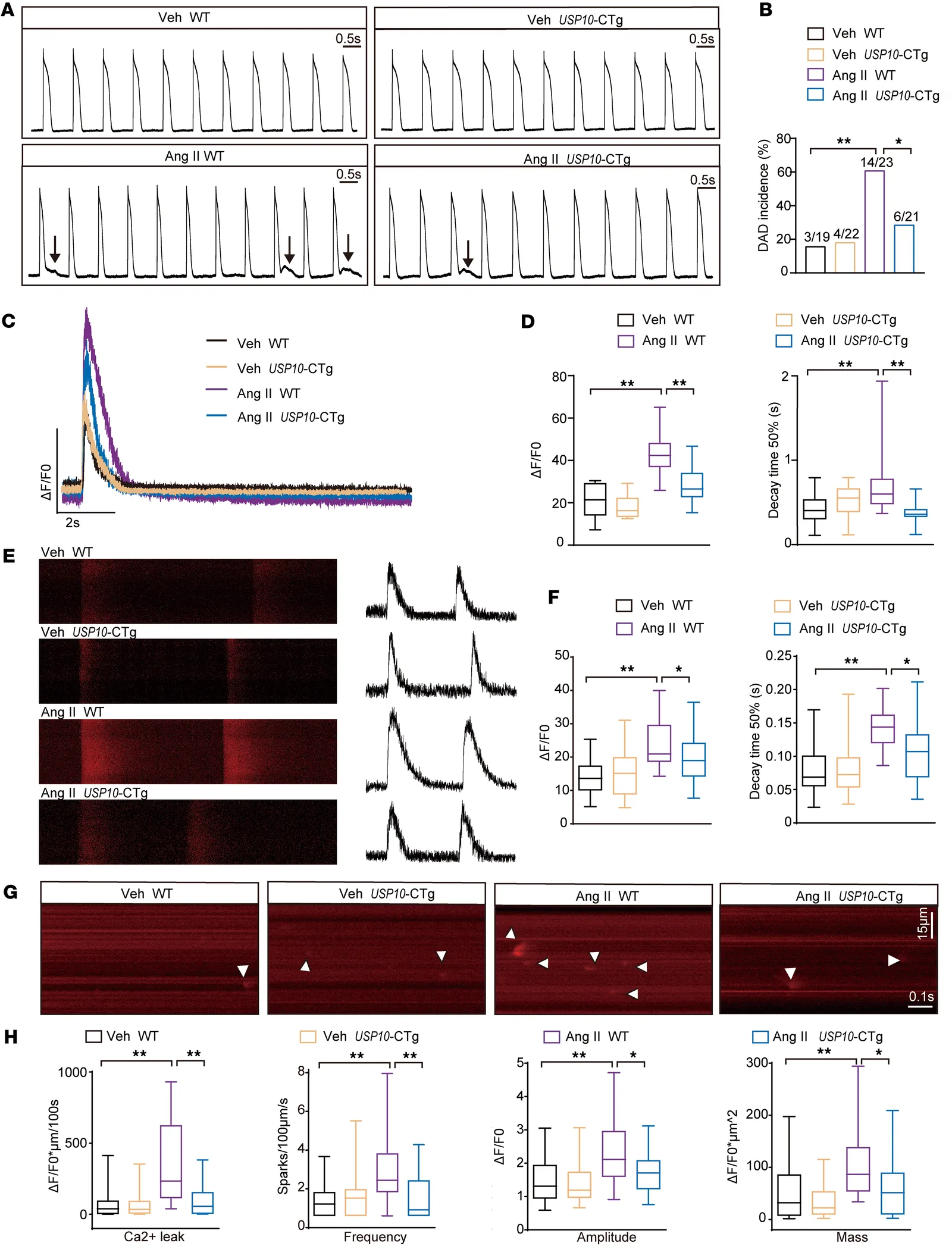
USP10 overexpression mitigates abnormal calcium handling and DADs. (**A**) Representative DAD images recorded from atrial cardiomyocytes obtained from WT and *Usp10*-CTg mice in response to vehicle or Ang II. (**B**) Statistical results of DADs in the indicated groups (*n* = 19–23/group). (**C**) Representative Ca^2+^ transients (CaTs) evoked by 10 mM caffeine in Ca^2+^-free conditions in atrial cells isolated from WT and *Usp10*-CTg mice treated with vehicle or Ang II. (**D**) Statistical results of CaT amplitudes in the indicated groups (*n* = 15–22/group). (**E**) Representative Ca²^+^ wave images in atrial cardiomyocytes from WT and *Usp10*-CTg mice, recorded following 1-Hz pacing after treatment with either vehicle or Ang II. (**F**) Statistical results of amplitude and 50% decay time of Ca^2+^ waves in the indicated groups (*n* = 22–37/group). (**G**) Representative Ca^2+^ spark images in atrial cardiomyocytes obtained from WT and *Usp10*-CTg mice in response to vehicle or Ang II. Scale bar: 0.1 seconds. (**H**) Statistical results of Ca^2+^ spark–induced Ca^2+^ leak, Ca^2+^ spark frequency, Ca^2+^ spark amplitude, and Ca^2+^ spark mass in the indicated groups (*n* = 26–31/group). Data are presented as mean ± SEM and were analyzed using the χ^2^ test (**B**) and 2-way ANOVA followed by Tukey’s multiple-comparisons test (**D**, **F**, and **H**). *P < 0.05; **P < 0.01. USP10, ubiquitin-specific peptidase 10; AF, atrial fibrillation; Ang II, angiotensin II; CaTs, Ca^2+^ transients; DAD, delayed afterdepolarization.

To assess the functional impact of USP10 overexpression on atrial myocytes, we examined calcium handling and proarrhythmic states using whole-cell patch-clamp techniques in atrial cells isolated from WT and *Usp10*-CTg mice treated with either vehicle or Ang II (22). USP10 overexpression significantly reduced the frequency of delayed afterdepolarizations (DADs) induced by Ang II (Figure 3, A and B), highlighting its protective role in maintaining atrial electrophysiological stability.

DADs are often associated with Ca^2+^-handling abnormalities that promote cellular triggered activity in AF. To evaluate the effect of USP10 on Ca^2+^ handling, we assessed caffeine-induced calcium transients (CaTs), an indicator of sarcoplasmic reticulum Ca²^+^ content. Sarcoplasmic reticulum Ca²^+^ homeostasis has a critical role in maintaining myocardial electrical stability (23). Ang II treatment increased the amplitude of caffeine-induced CaTs; however, USP10 overexpression reversed this increase compared with Ang II–treated cardiomyocytes (Figure 3, C and D). Following 1-Hz pacing, Ang II–treated atrial myocytes exhibited a higher susceptibility to spontaneous Ca^2+^ waves compared with vehicle controls (Figure 3E). Furthermore, the frequency of spontaneous Ca^2+^ waves was lower in USP10-overexpressing cardiomyocytes compared with Ang II–treated cells (Figure 3E). USP10 overexpression also reduced both the amplitude and 50% decay time of spontaneous Ca^2+^ waves (Figure 3F). Since spontaneous Ca^2+^ waves are often linked to enhanced sarcoplasmic reticulum Ca^2+^ release via RyR2 channels, we measured Ca^2+^ sparks as an indication of RyR2 function. USP10 overexpression significantly reduced Ca^2+^ spark–mediated leak, Ca^2+^ spark frequency, and Ca^2+^ spark mass (Figure 3, G and H). These findings suggest that USP10 overexpression ameliorates Ang II–induced abnormal calcium handling, thereby reducing AF susceptibility.

### USP10 contributes to the restoration of mitochondrial function.

**Figure 4:**
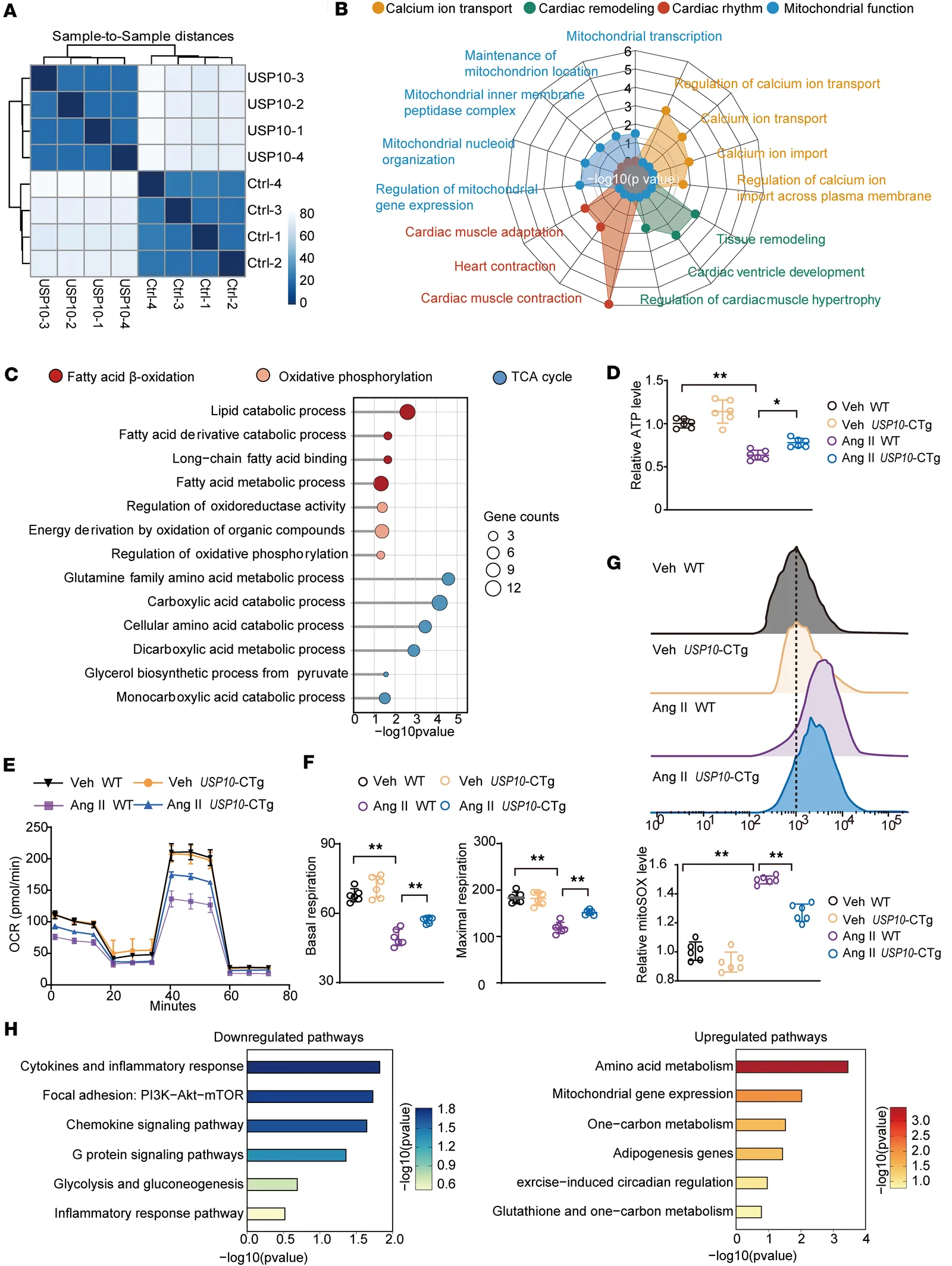
USP10 overexpression contributes to the restoration of mitochondrial function. (**A**) Sample-to-sample distance plot of HL-1 atrial myocytes expressing Ad-USP10 or Ad-Vector analyzed by RNA-seq (*n* = 4/group). (**B**) GO enrichment analysis showing enriched pathways related to mitochondrial function, calcium ion transport, and cardiac rhythm based on RNA-seq data (*n* = 4/group). (**C**) GO enrichment analysis showing enriched pathways related to fatty acid β-oxidation, oxidative phosphorylation, and the TCA cycle (*n* = 4/group). (**D**) ATP content in atrial cardiomyocytes obtained from WT and *Usp10*-CTg mice in response to vehicle or Ang II (*n* = 6/group). (**E**) OCR profiles indicating mitochondrial respiratory function in atrial cells isolated from WT and *Usp10*-CTg mice treated with vehicle or Ang II. (**F**) Quantification of basal respiration and maximal respiration in atrial cells isolated from WT and *Usp10*-CTg mice treated with vehicle or Ang II (*n* = 6/group). (**G**) MitoSOX-based flow cytometry of ROS in atrial cells isolated from WT and *Usp10*-CTg mice treated with vehicle or Ang II, combining a schematic illustration of the method with quantitative data (bottom; *n* = 6). (**H**) WikiPathways enrichment analysis of differentially expressed genes (DEGs) identified from RNA-seq by comparing Ang II–treated HL-1 cells expressing Ad-USP10 with those expressing Ad-Vector. Upregulated and downregulated DEGs were analyzed separately. Data are presented as mean ± SEM and were analyzed using 2-way ANOVA followed by Tukey’s multiple-comparison test (**D**, **F**, and **G**). *P < 0.05; **P < 0.01. USP10, ubiquitin-specific peptidase 10; Ang II, angiotensin II; GO, Gene Ontology; OCR, oxygen consumption rate; TCA cycle, tricarboxylic acid cycle; ROS, reactive oxygen species.

To elucidate the mechanisms by which USP10 regulates AF susceptibility, we performed RNA-seq on HL-1 cardiomyocytes infected with adenovirus carrying either *Usp10* (Ad-USP10) or vector DNA (Ad-Vector) and treated with Ang II. Sample-to-sample clustering analysis revealed significant transcriptional differences between USP10-overexpressing and control cells (Figure 4A). Gene Ontology (GO) analysis identified several AF-associated cellular processes affected by USP10 expression, including mitochondrial function, calcium ion transport, cardiac remodeling, and cardiac rhythm (Figure 4B). Given the critical role of mitochondrial function in AF initiation and progression, we further analyzed genes related to mitochondrial function affected by USP10 overexpression using pathway analysis. GO enrichment analysis showed significant enrichment of genes associated with fatty acid β-oxidation, oxidative phosphorylation, and the tricarboxylic acid (TCA) cycle following USP10 overexpression, suggesting broad effects on mitochondrial energy metabolism (Figure 4C).

To validate these findings, we measured ATP content in atrial cells isolated from WT and *Usp10*-CTg mice treated with vehicle or Ang II. As anticipated, ATP levels decreased in response to Ang II stimulation, but USP10 overexpression significantly restored ATP levels (Figure 4D). Furthermore, while Ang II stimulation reduced mitochondrial basal and maximal respiratory capacity, USP10 overexpression significantly enhanced mitochondrial respiratory function, as evidenced by an increased mitochondrial oxygen consumption rate (OCR) (Figure 4, E and F). Using MitoSOX-based flow cytometry, we assessed mitochondrial ROS production in atrial cells isolated from WT and *Usp10*-CTg mice treated with either vehicle or Ang II, and found that USP10 overexpression significantly suppressed mitochondrial ROS levels (Figure 4G).

We also investigated the global impact of USP10 on pathways related to cardiac remodeling and AF progression. Pathway enrichment analysis of differentially expressed genes (DEGs) identified by comparing Ang II–treated HL-1 cells expressing Ad-USP10 with those expressing Ad-Vector revealed that genes downregulated by USP10 overexpression were significantly enriched in pathways associated with cytokine and inflammatory responses, focal adhesion/PI3K/Akt/mTOR signaling, chemokine signaling, G protein signaling, and glycolysis/gluconeogenesis (Figure 4H). In contrast, genes upregulated in USP10-overexpressing cells were enriched in pathways involved in amino acid metabolism, mitochondrial gene expression, 1-carbon metabolism, adipogenesis-related genes, exercise-induced circadian regulation, and glutathione/1-carbon metabolism (Figure 4H). Collectively, these results suggest that USP10 contributes to reducing AF susceptibility, potentially by enhancing mitochondrial function.

### USP10 interacts with and deubiquitinates NDUFS1.

**Figure 5:**
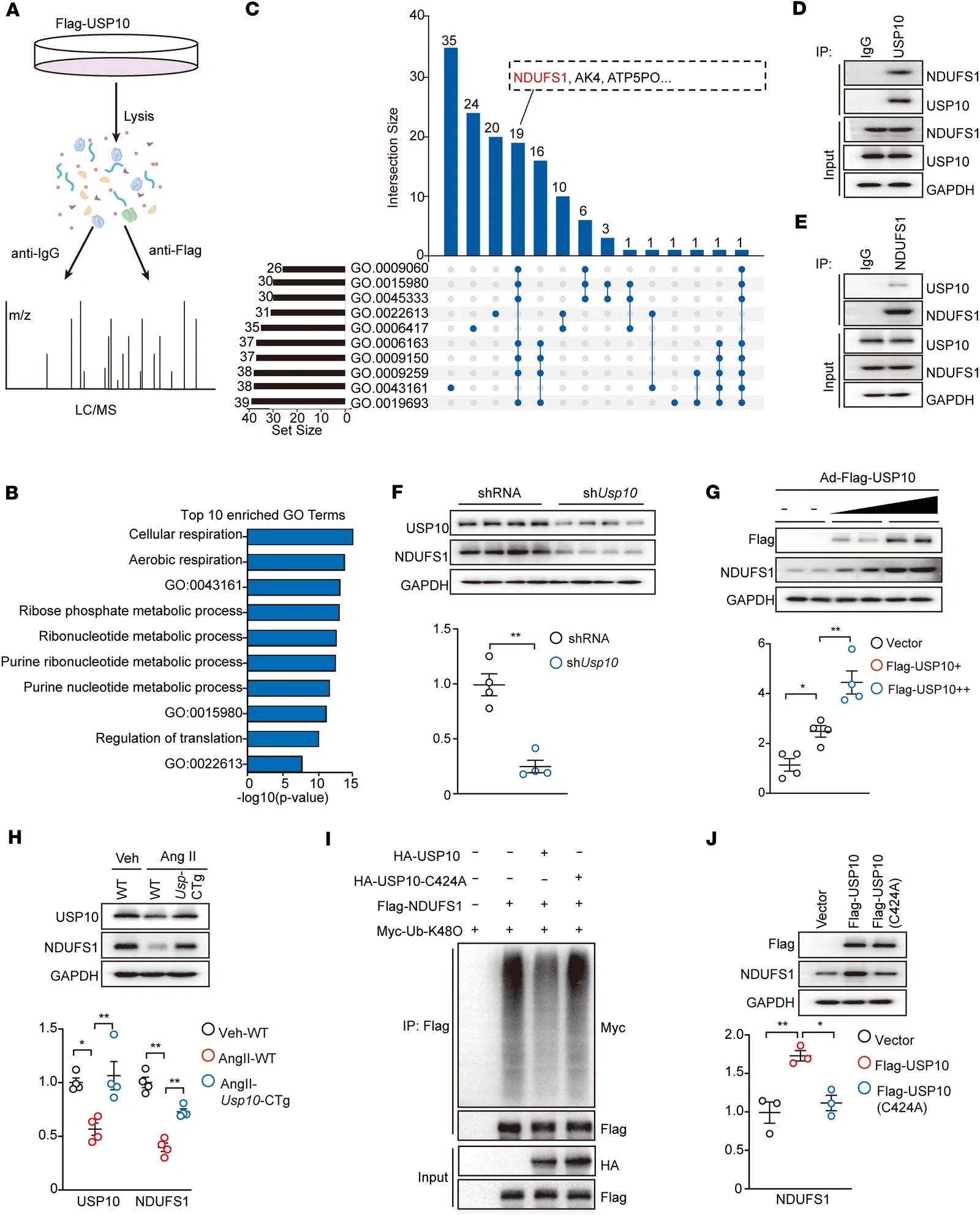
USP10 interacts with and deubiquitinates NDUFS1. (**A**) Diagram illustrating the experimental design used to explore USP10-interacting proteins by IP-MS in HL-1 cells. (**B**) GO analysis showing the top 10 enriched terms of USP10-binding proteins. (**C**) UpSet plot illustrating the intersections across different biological events of USP10-binding proteins. (**D** and **E**) Co-IP and Western blot analyses showing the interaction between USP10 and NDUFS1 in HL-1 cells. (**F**) Western blot analysis of USP10 and NDUFS1 protein levels in HL-1 atrial myocytes expressing Ad-sh*Usp10* or Ad-shRNA (*n* = 4/group). (**G**) Western blot analysis of USP10 and NDUFS1 protein levels in HL-1 atrial myocytes expressing different amounts of Ad-Flag-USP10 adenovirus (*n* = 4/group). (**H**) Western blot analysis of USP10 and NDUFS1 protein levels in mouse atrial tissues in the indicated groups (*n* = 4/group). (**I**) Representative Western blot images of K48-type ubiquitination of NDUFS1 in HL-1 cells in the indicated groups. (**J**) Western blot analysis of NDUFS1 protein levels in HEK293T cells expressing different plasmids (*n* = 3/group). Data are presented as mean ± SEM and were analyzed using an unpaired, 2-tailed Student’s *t* test (**F**) and 1-way ANOVA with Tukey’s post hoc test (**G**, **H**, and **J**). *P < 0.05; **P < 0.01. USP10, ubiquitin-specific peptidase 10; NDUFS1, NADH:ubiquinone oxidoreductase subunit S1; Ang II, angiotensin II; GO, Gene Ontology.

To uncover the underlying mechanisms by which USP10 influences mitochondrial function, we performed immunoprecipitation followed by mass spectrometry (IP-MS) to identify USP10-interacting proteins (Figure 5A). GO analysis of these interacting proteins revealed that many are associated with mitochondrial function, suggesting a close link between USP10 and mitochondrial regulation (Figure 5B). An UpSet plot analysis highlighted NDUFS1 as a key protein appearing in multiple intersections across various biological processes, indicating its involvement in USP10’s regulation of cellular functions (Figure 5C). NDUFS1, a crucial component of complex I in the mitochondrial electron transport chain, is essential for efficient energy production and mitochondrial function (24). Co-IP experiments confirmed the interaction between USP10 and NDUFS1 (Figure 5, D and E). Given that USP10 is a deubiquitinase known for regulating protein degradation by removing lysine 48–linked (K48-linked) ubiquitin from target proteins, we investigated the impact of USP10 on NDUFS1 protein levels. Both loss- and gain-of-function studies demonstrated that USP10 increases NDUFS1 protein expression (Figure 5, F and G). Furthermore, USP10 overexpression reversed the Ang II–induced reduction in NDUFS1 levels in mouse atrial tissues (Figure 5H). To further validate this mechanism, we used USP10-C424A, an enzymatically inactive mutant that lacks deubiquitinating activity (25). As shown in Figure 5I, while WT USP10 overexpression reduced K48-linked ubiquitination of NDUFS1, the USP10-C424A mutant had no effect on NDUFS1 ubiquitination. Consistently, Western blot analysis showed that the increase in NDUFS1 protein levels induced by USP10 overexpression was not observed in cells expressing USP10-C424A (Figure 5J). These findings identify NDUFS1 as a direct target of USP10 and suggest that NDUFS1 plays a critical role in mediating USP10’s regulatory effects on mitochondrial function.

### USP10 deubiquitinates NDUFS1 at K621.

**Figure 6:**
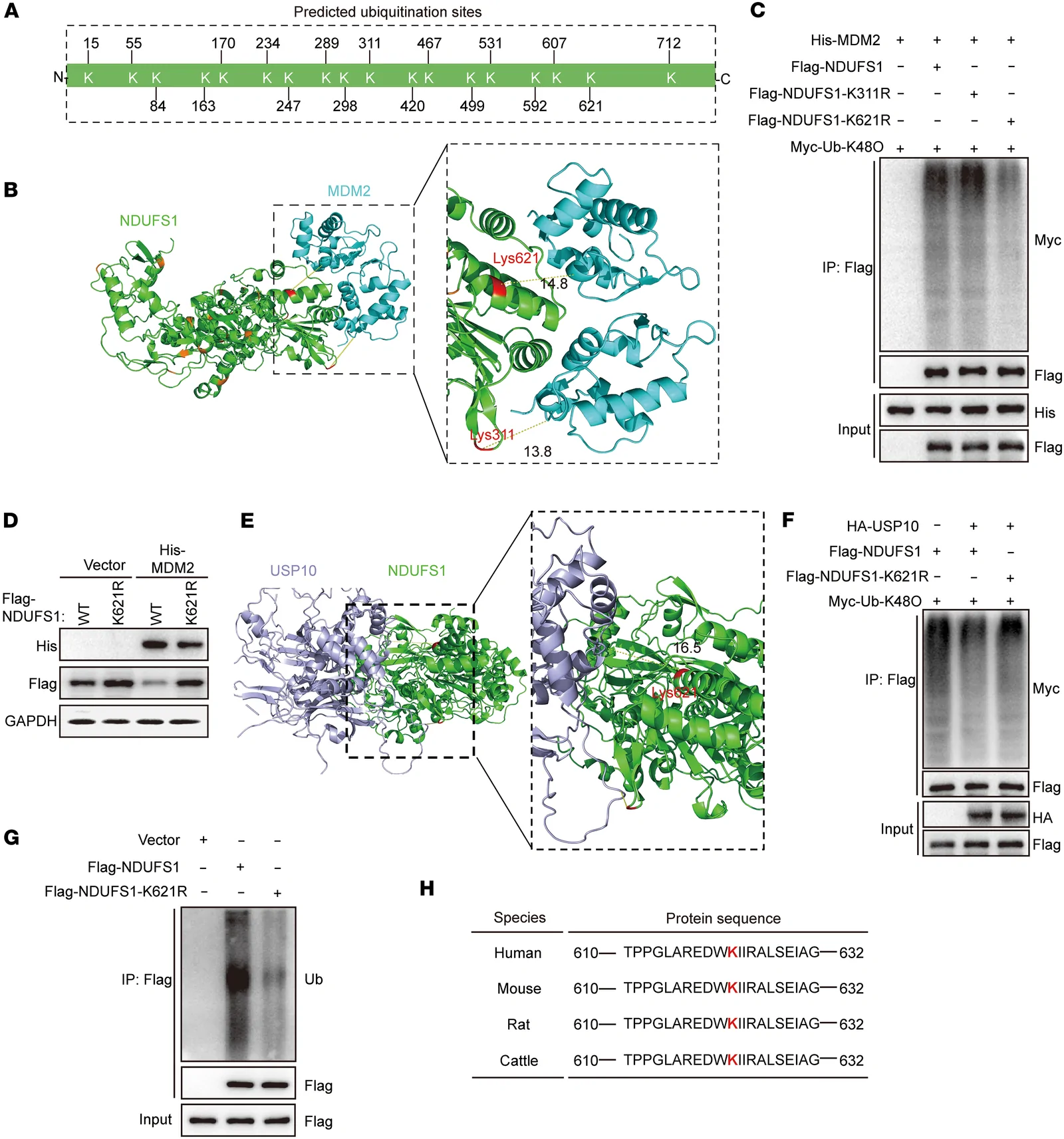
USP10 deubiquitinates NDUFS1 at K621. (**A**) Predicted ubiquitination sites of NDUFS1 using GPS-Uber. (**B**) Molecular docking simulation and calculation of the interaction sites between MDM2 (blue) and NDUFS1 (green). (**C**) Western blot analysis of K48-type ubiquitination of NDUFS1 in the indicated groups. (**D**) Representative Western blots showing Flag-NDUFS1 protein levels in the indicated groups. (**E**) Molecular docking simulation and calculation of the interaction sites between USP10 (purple) and NDUFS1 (green). (**F**) Western blot analysis of K48-type ubiquitination of NDUFS1 in the indicated groups. (**G**) Western blot analysis of the ubiquitination of WT and K621R mutant immunoprecipitated with NDUFS1. (**H**) Alignment of K621 and adjacent amino acids of NDUFS1 across different species. USP10, ubiquitin-specific peptidase 10; NDUFS1, NADH:ubiquinone oxidoreductase subunit S1.

Following the identification of NDUFS1 as a direct target of USP10, we further investigated the specific ubiquitination sites on NDUFS1. Using GPS-Uber, a widely used ubiquitination prediction tool (26), we identified multiple potential ubiquitination sites on NDUFS1 (Figure 6A). Previous studies have demonstrated that MDM2, an E3 ubiquitin ligase, interacts with NDUFS1 (27). We analyzed the spatial relationship between MDM2 and the predicted ubiquitination sites on NDUFS1, focusing specifically on those sites in close proximity to MDM2. The predicted docking models identified 2 key interaction sites — Lys311 (K311) and Lys621 (K621) — between NDUFS1 and MDM2, while other predicted ubiquitination sites were located farther away (Figure 6B). To experimentally validate these ubiquitination sites, we generated NDUFS1-K311R and NDUFS1-K621R mutants by substituting lysine (K) with arginine (R) at positions 311 and 621, respectively. As shown in Figure 6C, the ubiquitination level of NDUFS1-K311R was similar to that of WT NDUFS1. In contrast, the ubiquitination level of NDUFS1-K621R was significantly reduced, demonstrating that MDM2 specifically ubiquitinates NDUFS1 at K621. Consistently, NDUFS1 expression was reduced in MDM2-transfected cells, but MDM2 did not further decrease the protein level of NDUFS1-K621R mutant (Figure 6D).

To confirm that USP10 deubiquitinates NDUFS1 specifically at K621, we assessed the spatial interaction between USP10 and NDUFS1. Predicted docking models revealed a direct interaction between USP10 and NDUFS1 at K621 (Figure 6E). Supporting this, a ubiquitination assay demonstrated that USP10 was unable to further reduce the ubiquitination level of NDUFS1-K621R compared with WT NDUFS1 (Figure 6F). Ubiquitination analysis of immunoprecipitated NDUFS1 (WT and K621R mutant) confirmed that K621 is the primary ubiquitination site (Figure 6G). Notably, the K621 ubiquitination site and the surrounding protein sequence are conserved among various vertebrates, including humans, mice, rats, and cattle (Figure 6H).

### NDUFS1-K621R attenuates abnormal calcium handling, DADs, and AF susceptibility.

To directly assess the functional role of NDUFS1 in AF, we generated adeno-associated virus–mediated (AAV-mediated) NDUFS1 and NDUFS1-K621R overexpression mouse models and evaluated Ca^2+^ handling, proarrhythmic activity, and AF susceptibility.

NDUFS1 overexpression significantly reduced Ang II–induced Ca²^+^ sparks, calcium leak levels, and spark area, whereas the K621R variant produced a further reduction in these parameters (Supplemental Figure 1, A and B; supplemental material available online with this article; https://doi.org/10.1172/jci.insight.195761DS1). Consistently, NDUFS1 overexpression decreased the frequency of DADs under Ang II stimulation, with a more pronounced effect observed in the K621R group (Supplemental Figure 1, C and D). In vivo, both NDUFS1 and its K621R variant attenuated Ang II–induced atrial fibrosis (Supplemental Figure 1, E and F) and reduced AF incidence and duration (Supplemental Figure 1, G and H), with the K621R variant consistently exhibiting a stronger effect across all assays.

Collectively, these findings demonstrate that NDUFS1 exerts a protective effect against Ang II–induced atrial remodeling and arrhythmogenesis, and that the K621R mutation enhances its functional activity.

### USP10 knockdown exacerbates abnormal calcium handling, DADs, and AF susceptibility.

**Figure 7:**
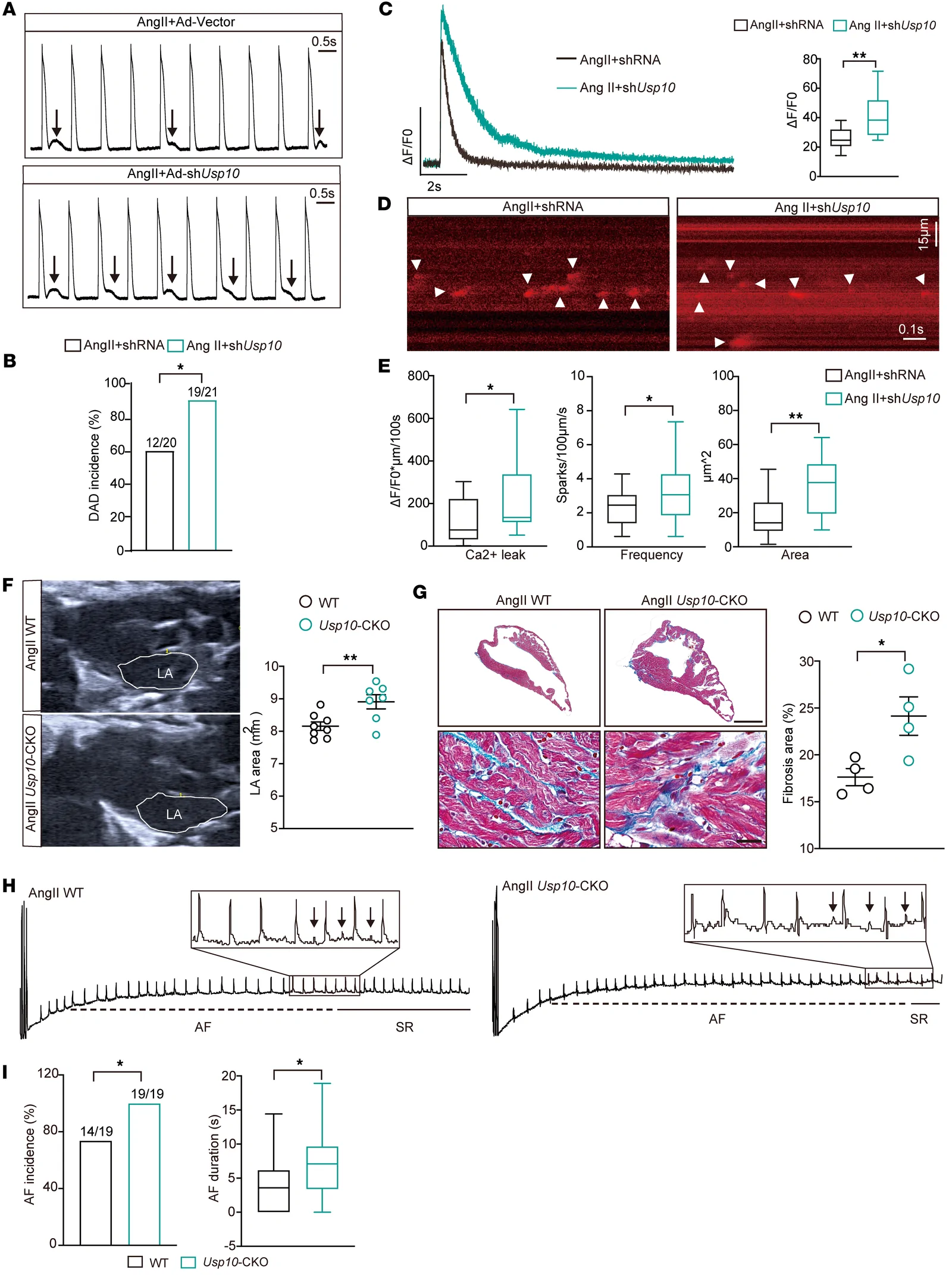
USP10 knockdown exacerbates abnormal calcium handling, DADs, and AF susceptibility. (**A**) Representative DAD images recorded from HL-1 cells expressing Ad-shRNA or Ad-sh*Usp10* under Ang II treatment. (**B**) Statistical results of DAD incidence. (**C**) Representative Ca^2+^ transient (CaT) images and statistical results of CaT amplitudes in the indicated groups (*n* = 15–22/group). (**D**) Representative Ca^2+^ spark images obtained from HL-1 cells expressing Ad-shRNA or Ad-sh*Usp10* in response to Ang II stimulation. Scale bar: 0.1 seconds. (**E**) Statistical results of Ca^2+^ spark–induced Ca^2+^ leak, Ca^2+^ spark frequency, and Ca^2+^ spark area in the indicated groups (*n* = 20–31/group). (**F**) Representative echocardiography images and quantification of left atrial size from WT and *Usp10*-CKO mice under Ang II treatment (*n* = 7–8/group). (**G**) Representative images and quantitative results of Masson’s trichrome staining on left atrial sections of *Usp10*-CKO and WT control mice in response to Ang II treatment (*n* = 4/group). (**H**) Representative limb lead ECG traces recorded from *Usp10*-CKO and WT control mice in response to Ang II treatment. (**I**) Fraction of mice with successful AF induction relative to the total number and AF duration in *Usp10*-CKO and WT control mice in response to Ang II treatment (*n* = 19/group). Data are presented as mean ± SEM and were analyzed using the χ^2^ test (**B** and **I** [left]) and the 2-tailed Student’s *t* test (**C**, **E**–**G**, and **I** [right]). *P < 0.05; **P < 0.01. NS, not significantly different. USP10, ubiquitin-specific peptidase 10; AF, atrial fibrillation; Ang II, angiotensin II; CaTs, Ca^2+^ transients; DAD, delayed afterdepolarization; ECG, electrocardiogram; CKO, cardiomyocyte-specific knockout.

Given that USP10 overexpression mitigates abnormal calcium handling, DADs, atrial remodeling, and AF susceptibility in mice, we hypothesized that USP10 knockdown would have the inverse effects. To test this hypothesis, we performed a series of loss-of-function experiments. Consistent with our expectations, USP10 knockdown promoted DADs, as evidenced by an increased frequency (Figure 7, A and B). Additionally, USP10 knockdown significantly increased the amplitude of Ang II–induced CaTs (Figure 7C) and the incidence of Ca^2+^ sparks (Figure 7, D and E).

To further assess the impact of *Usp10* loss, we utilized cardiomyocyte-specific *Usp10*-knockout (*Usp10*-CKO) mice to evaluate whether *Usp10* KO would exacerbate Ang II–induced atrial remodeling and AF susceptibility. As anticipated, *Usp10* KO significantly amplified Ang II–induced left atrial enlargement, atrial fibrosis, and the incidence and duration of AF (Figure 7, F–I). Collectively, these findings demonstrate that the loss of *Usp10* exacerbates Ang II–induced abnormal calcium handling, DADs, and AF susceptibility, highlighting the critical role of USP10 in maintaining normal electrophysiological and structural integrity.

## Discussion

Despite advances in our understanding of AF, the intricate mechanisms driving its pathogenesis remain incomplete, limiting the development of targeted and effective therapies. This study uncovers a critical role for USP10 in AF and highlights it as a potential therapeutic target. Our findings demonstrate that USP10 expression is markedly reduced in atrial tissues from patients with AF and Ang II–treated mouse models. USP10 overexpression mitigates key arrhythmogenic factors, including aberrant calcium handling and DADs, which are critical contributors to AF pathogenesis. Specifically, USP10 overexpression attenuates Ang II–induced abnormal calcium waves and decreases the frequency of DADs, thus reducing cellular proarrhythmic states. Additionally, USP10 enhances mitochondrial oxidative phosphorylation, alleviating mitochondrial dysfunction, a critical contributor to AF progression. Mechanistically, USP10 interacts with and stabilizes NDUFS1, a key subunit of complex I in the mitochondrial electron transport chain, by deubiquitinating it at K621. Our findings position USP10 as a previously unrecognized modulator of mitochondrial function and calcium handling in AF.

AF is frequently linked to atrial remodeling, which is characterized by cardiomyocyte hypertrophy and fibrosis (28). These structural changes contribute to triggered activity and electrical re-entry, thus perpetuating AF episodes (29, 30). Our study demonstrates that USP10 overexpression effectively attenuates Ang II–induced atrial remodeling and reduces AF susceptibility. Importantly, the protective effects of USP10 extend beyond structural alterations; USP10 also addresses aberrant Ca^2+^ handling. Proper Ca^2+^ handling is essential for maintaining cardiac rhythm, and its disruption promotes AF triggers and forms a substrate for arrhythmia (31–33). Our findings confirm that USP10 overexpression reverses Ang II–induced abnormalities in triggered activity and CaT kinetics, underscoring its critical role in protecting against atrial electrical instability and maladaptive remodeling.

Our study further highlights the crucial role of USP10 in mitigating mitochondrial dysfunction, a pivotal factor in the progression of AF (4). Mitochondrial dysfunction leads to reduced ATP production, exacerbating energy deficiency in atrial myocytes and contributing to contractile dysfunction and electrical instability (34). Insufficient ATP levels impact ATP-sensitive ion channels and Ca^2+^ transport systems, including the sarco/endoplasmic reticulum calcium ATPase (SERCA) pump, resulting in defective Ca^2+^ reuptake and elevated cytosolic Ca^2+^ levels (35, 36). Our findings demonstrate that USP10 restores calcium handling, enhances mitochondrial oxidative phosphorylation, and increases ATP production. Moreover, mitochondrial dysfunction is associated with increased oxidative stress and cytokine release, which further promotes atrial remodeling (34). By restoring mitochondrial function, USP10 alleviates these deleterious effects, ultimately helping to prevent atrial structural changes associated with AF.

Based on current evidence, mitochondrial dysfunction likely represents a bidirectional factor in AF pathophysiology — both a contributor to AF susceptibility/progression and a consequence of AF (34, 37). Notably, mitochondrial dysfunction is a well-established driver of adverse cardiac remodeling, and maladaptive remodeling is a recognized substrate promoting AF initiation and maintenance. Importantly, our data support the notion that USP10 alleviates AF initiation and maintenance by improving mitochondrial function, through deubiquitination and stabilization of NDUFS1, thereby preserving mitochondrial bioenergetics and attenuating remodeling-associated AF vulnerability. Collectively, these findings suggest that USP10 plays a protective role in reducing AF susceptibility and maintenance.

To elucidate the molecular mechanisms by which USP10 regulates mitochondrial function, we performed co-IP assays coupled with liquid chromatography–tandem MS (LC-MS/MS) analysis, identifying NDUFS1 as a direct target of USP10. NDUFS1, a core subunit of mitochondrial complex I, plays a critical role in controlling complex I activity and mitochondrial oxidative phosphorylation (24). Our data reveal that USP10 deubiquitinates NDUFS1, preventing its degradation and enhancing mitochondrial function. Although previous studies suggested that MDM2 binds to NDUFS1 (27), our study establishes MDM2 as a direct E3 ligase for NDUFS1, promoting K48-linked ubiquitination at K621. USP10 counteracts this by specifically removing K48-linked ubiquitin from NDUFS1 at K621, thereby protecting it from proteasomal degradation and maintaining mitochondrial integrity.

A previous study reported that increased USP10 in ventricular cardiomyocytes mitigates pathological cardiac hypertrophy, at least in part, through deubiquitination of SIRT6 (16). In the present study, however, we focused on atrial cardiomyocytes and found that USP10 expression was reduced in this context. Importantly, our RNA-seq analysis indicated that the predominant pathways associated with USP10 in atrial myocytes were related to mitochondrial function and energy metabolism. Consistently, our IP-MS data further identified NDUFS1 as a key USP10-interacting/regulatory target, supporting a USP10/NDUFS1 mitochondrial axis as a major mechanism in atrial remodeling and AF susceptibility. We agree that USP10 may exert context-dependent and multifaceted regulatory roles in cardiovascular diseases. Nevertheless, based on our data, we propose that the USP10/NDUFS1 pathway plays a protective role in alleviating atrial remodeling and AF initiation/maintenance. We propose that this pathway contributes to AF pathogenesis without claiming that the USP10/NDUFS1 mechanism explains the full etiology of AF.

In summary, this study provides previously unrecognized insights into the pathophysiology of AF and mitochondrial dysfunction. Our findings demonstrate that upregulation of USP10 ameliorates mitochondrial dysfunction and restores calcium handling, addressing key mechanisms underlying AF progression. By stabilizing NDUFS1 and enhancing mitochondrial oxidative phosphorylation, USP10 modulation offers a potential strategy to mitigate atrial remodeling, electrical instability, and AF susceptibility. These results underscore the therapeutic potential of enhancing USP10 activity to prevent AF progression.

## Methods

### Sex as a biological variable.

Both male and female mice were included in this study, and experiments were conducted using sex-balanced cohorts across all experimental groups. However, due to the limited sample size within each sex, the study was underpowered to detect sex-specific differences, and no definitive conclusions regarding sexual dimorphism can be drawn.

In addition, the sex distribution of human right atrial appendage samples obtained from patients with AF and SR controls is provided in Supplemental Table 1. Both male and female patients were included in both groups; however, due to the limited sample size, sex-stratified analyses were not performed.

### Human atrial samples.

Human right atrial appendages were collected from patients undergoing open-heart surgery for valve replacement or coronary bypass grafting at the First Affiliated Hospital of Zhengzhou University and informed written consent was given prior to the inclusion of patients in the study. Patients were divided into 2 groups based on cardiac rhythm: the normal SR group and the persistent AF group. Detailed baseline characteristics of the patient cohort are provided in Supplemental Table 1.

### Animals.

*Usp10*-CTg genetically modified mice were obtained as described previously (16). Briefly, *Usp10*-CTg mice were generated by cloning the full-length mouse *Usp10* cDNA downstream of the cardiac α-MHC promoter. *Usp10*-CKO mice were created by crossing *Usp10^fl/fl^* mice with α-MHC-MerCreMer mice. To induce cardiomyocyte-specific *Usp10* deletion, 6- to 8-week-old *Usp10*-CKO mice with matched sex were administered tamoxifen for 5 consecutive days. Following a washout period of 2 weeks, during the 8- to 10-week age window, the mice underwent a 4-week disease modeling phase wherein they received continuous infusion of either saline or Ang II (1600 ng/kg/min) via osmotic minipumps. All mice were maintained on a 12-hour light/12-hour dark cycle with ad libitum access to food and water.

### Animal protocols.

AF was induced in mice as previously described (38). Sex-matched mice were anesthetized with 1% sodium pentobarbital and placed on a heating pad. A 4.5-F mouse esophageal electrophysiology catheter (Kardiotek Biomedical Technologies) was placed in the esophagus near the left atrium. The catheter was connected to an external stimulator (Dual-channel Programmable Electric Stimulator, Kardiotek Biomedical Technologies). Lead II surface electrocardiograms (ECGs) were recorded using a bioamplifier (PowerLab 8/35, AD Instruments). Inducibility of atrial arrhythmias was tested by applying a burst-pacing protocol with 5 decremental cycle lengths (50, 40, 30, 25, and 20 ms), each consisting of 15-second stimulus trains followed by 3-minute interburst intervals. AF was defined as rapid, irregular atrial rhythm with irregularly irregular R-R intervals lasting at least 1 second. All ECG data were analyzed using LabChart Pro v8.1.5 software (AD Instruments). AF susceptibility was evaluated by the AF incidence (proportion of mice with AF episodes) and AF average duration. The experiments were performed in a blinded manner.

### Recombinant AAV9 vectors.

Recombinant cardiotropic AAV9 vectors carrying NDUFS1 (pcAAV-cTNT-Ndufs1-3xFLAG-P2A-ZsGreen1-tWPA), NDUFS1-K621R (pcAAV-cTNT-Ndufs1(p.K621R)-3xFLAG-P2A-ZsGreen1-tWPA), or empty vector under the control of the cTnT promoter were constructed by Obio Technology Co., Ltd. Six-week-old C57BL/6 mice were administered AAV9 (1 × 10¹¹ viral particles per mouse) via tail vein injection. When the mice reached 8 weeks of age, they entered a 4-week disease modeling phase during which they received continuous infusion of either saline or Ang II (1600 ng/kg/min) via osmotic minipumps.

### Echocardiography.

Echocardiography was conducted as previously described (39). Mice were anesthetized with 1% isoflurane, and echocardiographic measurements of the left atrial diameter and area were measured from the parasternal long-axis view using a Vevo2100 system equipped with a 17 MHz transducer (VisualSonics).

### Masson’s trichrome staining.

Heart samples were fixed with 4% paraformaldehyde, embedded in paraffin, sectioned at 5 μm thickness, and mounted on positively charged slides. Sections were stained using Masson’s trichrome stain according to standard protocols to visualize collagen fibers. Collagen volume was quantified using ImageJ software (version 1.54, NIH) by measuring the ratio of the blue-stained area to the total left atrial area.

### Cell culture and transfection.

HEK293T cells (ViCell, RRID: CVCL_0063) and HL-1 cells (SUNNCELL, RRID: CVCL_0303) were cultured in DMEM (Gibco, 11965092) or Claycomb medium (Sigma-Aldrich, 51800C), respectively, supplemented with 10% FBS (Gibco, A5256801) and 1% penicillin-streptomycin (Gibco, 15140122) in a 5% CO_2_ atmosphere at 37°C. For overexpression experiments, HEK293T cells were transfected using Lipofectamine 2000 (Invitrogen, 11668019) and HL-1 cells were infected with adenovirus at a multiplicity of infection (MOI) of 50.

### OCR assay.

Primary mouse atrial cardiomyocytes were plated at a density of 5 × 10^4^/well in Seahorse XF plates. Baseline OCR was measured, followed by sequential injection of 1 μM oligomycin, 1 μM FCCP, and 0.5 μM rotenone/antimycin A to assess mitochondrial respiratory function. OCR data were analyzed using Seahorse XF software (Agilent).

### Mitochondrial ROS detection.

Cells were harvested by trypsinization, resuspended at 1 × 10^6^ cells/mL in fresh culture medium, and stained with MitoSOX Red reagent (Beyotime, S0061S) at a final concentration of 5 μM. After a 20-minute incubation at 37°C in the dark, cells were washed twice with PBS and analyzed using a flow cytometer (CytoFLEX, Beckman Coulter) with the PE channel configured for MitoSOX Red fluorescence.

### Western blotting.

Atrial tissues or cells were lysed in RIPA lysis buffer containing 0.1% protease inhibitor cocktail (Thermo Fisher Scientific, A32965). Protein concentrations were quantified using a BCA Protein Assay Kit (Thermo Fisher Scientific, A55861). Equal amounts of protein were separated by 10% SDS-PAGE and transferred to PVDF membranes (Thermo Fisher Scientific, 88518). Membranes were blocked with 5% skim milk and then incubated with primary antibodies followed by HRP-conjugated secondary antibodies. Immunoblots were visualized using an Enhanced Chemiluminescence (ECL) kit (Thermo Fisher Scientific, 32209). Detailed information on all antibodies used in this study is provided in Supplemental Table 2.

### IP and LC-MS/MS.

Cells were lysed in IP buffer (20 mM Tris-HCl, 150 mM NaCl, 1% NP-40, 1 mM EDTA) with protease inhibitors. After sonication and centrifugation, the supernatants were incubated with 2 μg of primary antibody specific for the target protein. Dynabeads Protein G beads (Invitrogen, 10004D) were added, and the mixture was incubated overnight at 4°C. The beads were washed, and proteins were eluted with elution buffer. Eluted samples were analyzed by Western blotting or LC-MS/MS.

For LC-MS/MS analysis, samples were processed using an LC-MS/MS system (Orbitrap Fusion Lumos, Thermo Fisher Scientific). LC and MS conditions were as follows: ion source at 2 kV, MS1 mass spectrometer scanning range of 350–1,500 *m*/*z*; resolution of 60,000; MS2 starting *m*/*z* fixed at 100; and resolution of 15,000.

### RT-qPCR.

Total RNA was extracted using TRIzol reagent (Invitrogen, 15596026) and reverse transcribed to cDNA using the PrimeScript RT reagent kit (TaKaRa, RR037A). Quantitative PCR (qPCR) was performed with SYBR Green Master Mix (Applied Biosystems, A46109). Relative mRNA levels of target genes were quantified using the 2^–ΔΔCt^ method with GAPDH as the internal control. Detailed information on primer sequences used in this study is provided in Supplemental Table 3.

### RNA-seq and DEG analysis.

RNA-seq and data analysis were performed as previously described (40). Total RNA was extracted from HL-1 cells using TRIzol reagent and subjected to sequencing on an Illumina NovaSeq X Plus platform by Novogene Co., Ltd. Clean reads were aligned to the mouse reference genome using HISAT2 (http://daehwankimlab.github.io/hisat2/). Differential gene expression analysis was conducted using the DESeq2 package (https://bioconductor.org/packages/DESeq2) in R (version 4.2.2; https://www.r-project.org/), with thresholds of adjusted *P* < 0.05 and |log2(fold change)| > 1.5 to identify significant DEGs. Pathway enrichment analysis was carried out using R (version 4.2.2).

### Calcium imaging.

Calcium signals were recorded using an inverted confocal microscope (Zeiss LSM 980). Cardiomyocytes were loaded with 5 μmol/L Cal-630 AM (Shanghai Maokang Biotechnology) and incubated at 37°C for 1 hour, followed by 30 minutes at room temperature. The dye was replaced with 1.8 mM Ca^2+^ Tyrode solution, and fluorescence was excited with a 633 nm laser using a 20× objective lens. Line scan images were captured at a sampling frequency of 1.23 ms per row, and CaTs were analyzed using ImageJ software. Change in fluorescence intensity (change in fluorescence divided by baseline fluorescence, ΔF/F0) was calculated as ([*F_t_* – *F_min_*]/*F_min_*) × 100%, where *F_t_* is the peak fluorescence intensity and *F_min_* is the minimum fluorescence intensity during the transients.

To analyze calcium sparks, cardiomyocytes loaded with Cal-630 AM were imaged using a 63× oil immersion lens. Line scan images were recorded at a sampling frequency of 1.23 ms per row, each containing 512 pixels. Calcium spark frequency and amplitude were analyzed using the “SparkMaster” plugin in ImageJ software, with amplitude expressed as ΔF/F0.

### Patch clamp.

Whole-cell patch-clamp recordings were performed using an Axopatch 200B amplifier (Molecular Devices) at room temperature. Pipettes, pulled from borosilicate glass with resistances of 2–4 MΩ using a P-97 puller (Sutter Instrument), were filled with an intracellular solution containing 135 mM K-gluconate, 10 mM KCl, 10 mM HEPES, 5 mM Mg-ATP, 0.3 mM Na_2_-GTP, and 1 mM EGTA 1 (pH adjusted to 7.2 with KOH).

To assess DADs, cells were stimulated using 5-ms, 900-mV square-wave pulses at varying pacing frequencies (0.5, 1, 2, and 5 Hz). After 10 seconds of steady-state pacing, the stimulation was halted, and afterdepolarizations, along with triggered activities, were recorded. DADs were identified as low-amplitude depolarizations that exceeded the diastolic membrane potential by more than 2 mV.

### Statistics.

All data are presented as mean ± SEM. Statistical analyses were performed using SPSS 19.0 software (IBM). Comparisons between 2 groups were conducted using a 2-tailed Student’s *t* test. For comparisons among multiple groups, 1-way ANOVA was used, followed by Tukey’s multiple-comparison test for equal variances or Tamhane’s T2 test when variances were not equal. Two-way ANOVA followed by Tukey’s multiple-comparison test was used for analyses involving 2 independent variables. The χ^2^ test was used for categorical data. A *P* value of less than 0.05 was considered statistically significant. In all box-and-whisker plots, the bounds of the boxes indicate the 25th and 75th percentiles, the line within each box indicates the median, and the whiskers extend to the minimum and maximum values. All data points are included, with no outliers excluded.

### Study approval.

All human studies were approved by the Ethics Committee of The First Affiliated Hospital of Zhengzhou University. All participants provided written informed consent. All animal procedures were approved by the Animal Care and Use Committee of The First Affiliated Hospital of Zhengzhou University (approval number 2021-KY-0940-002) and were conducted per the NIH *Guide for the Care and Use of Laboratory Animals* (National Academies Press, 2011).

### Data availability.

All data supporting the findings of this study are available within the article and its supplemental material. Values for all data points in graphs are reported in the Supporting Data Values file. Additional data are available from the corresponding author upon reasonable request. The RNA-seq data generated in this study have been deposited in the NCBI GEO under accession number GSE335617.

## Author contributions

WF, XXT, and GJZ conceived the idea and designed the experiments. WF, XXT, JZ, HL, LL, TYWW, and ZW conducted the experiments and analyzed the data. YXH performed the RNA-seq data analyses. WF prepared the figures. GJZ wrote the manuscript. All authors revised and approved the final manuscript.

## Conflict of interest

The authors have declared that no conflict of interest exists.

## Funding support

- Postdoctoral Fellowship Program of CPSF (GZC20241551).
- Fellowship of China Postdoctoral Science Foundation (2022T150593).

## Supplementary Material

Supplemental data

Unedited blot and gel images

Supporting data values

## Version 1 08/24/2026

Electronic publication
