# Supplementary material for: USP10 mitigates Ang II–induced atrial remodeling and atrial fibrillation susceptibility by deubiquitinating NDUFS1
Source: JCI Insight. 2026 Aug 24;11(16):e195761. doi: 10.1172/jci.insight.195761 (PMC13502181; doi:10.1172/jci.insight.195761)
Supplement: Supplementary file 1 [file jciinsight-11-195761-s338.pdf]

Supplemental Figures and Tables

USP10 Mitigates Ang II-induced Atrial Remodeling and Atrial Fibrillation Susceptibility by De-ubiquitinating NDUFS1

Wanrong Fu[1*], Xiao-Xu Tian[1*], Jianghua Zhou[2*], Yu-Xu Huang[3], Huan Li[1], Zhenya Wang[1], Tong-You Wade Wei[5], Li Li[4], Guo-Jun Zhao[1#]

1, Department of Cardiology, The First Affiliated Hospital of Zhengzhou University, Zhengzhou, 450052, China.

2, Department of Cardiology, The First Affiliated Hospital of Wenzhou Medical University, Wenzhou, China.

3, Department of Medicine, JingGangShan University, Ji'an, China.

4, Department of Cardiovascular Surgery, The First Affiliated Hospital of Zhengzhou University, Zhengzhou, China

5, Division of Cardiology, Department of Medicine, University of California, San Diego, La Jolla, California, USA

[*]These authors contributed equally to this study.

#Corresponding author:

Guo-Jun Zhao, MD & PhD, Department of Cardiology, The First Affiliated Hospital of Zhengzhou University, Zhengzhou, China. Email: zhaoguojun@zzu.edu.cn

Supplemental Figure 1

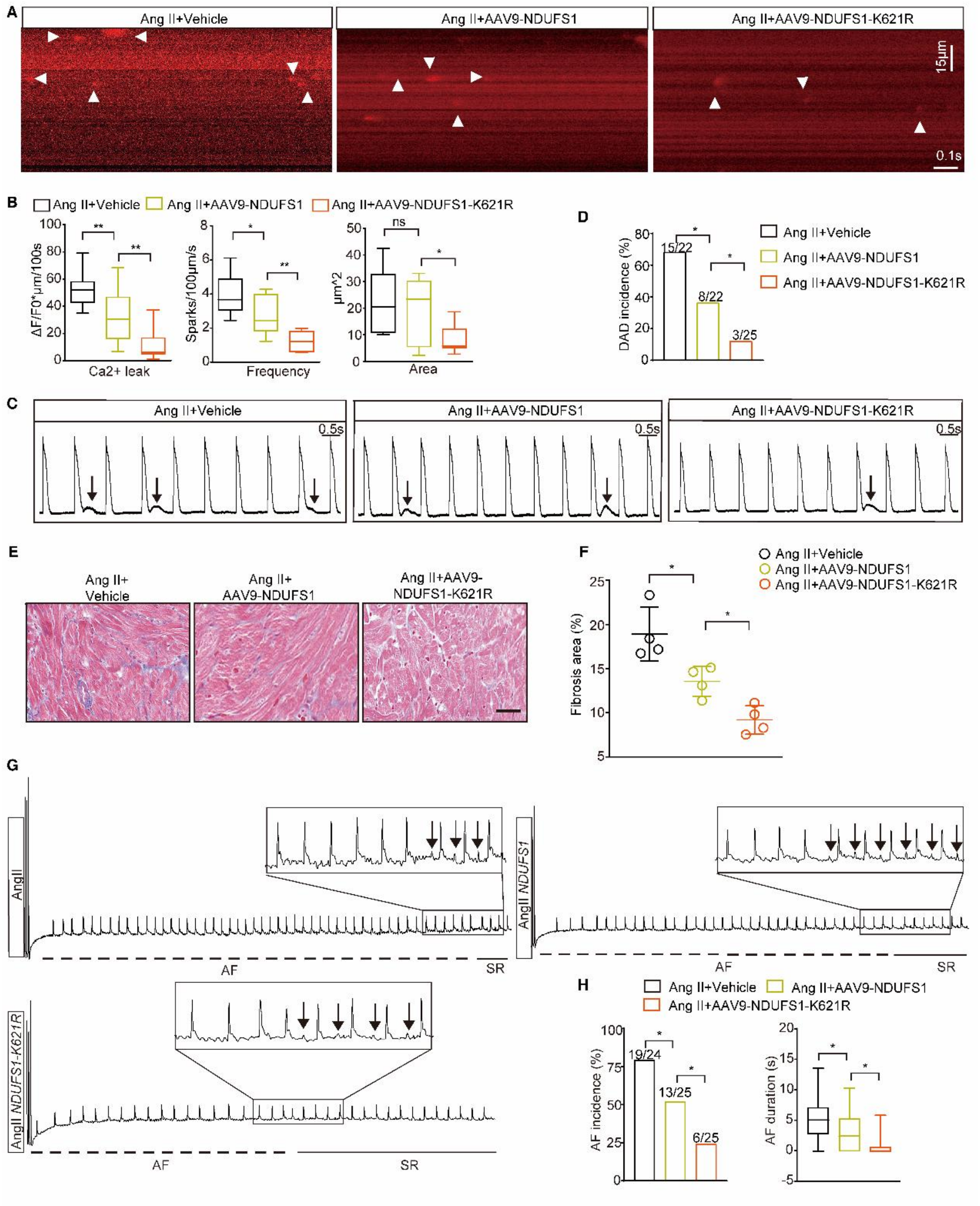

Figure S1. NDUFS1-K621R attenuates abnormal calcium handling, DADs, and AF susceptibility. A, Representative Ca^{2+} spark images obtained from adult mouse atrial cardiomyocytes with AAV-mediated overexpression of NDUFS1 or NDUFS1-K621R in response to Ang II stimulation; **B**, Statistical results of Ca^{2+}

spark-induced Ca^{2+} leak, Ca^{2+} spark frequency, Ca^{2+} spark area in the indicated groups (n=11/group); **C**, Representative DAD recordings in adult mouse atrial cardiomyocytes under Ang II treatment, comparing control and AAV-mediated NDUFS1 or NDUFS1-K621R overexpression groups; **D**, Statistical results of DADs incidence; **E** and **F**, Representative images and quantification of Masson's trichrome staining in left atrial sections from NDUFS1 and NDUFS1-K621R mice treated with Ang II (n=4/group); scale bars, 100 μm; **G**, Representative limb lead ECG traces from NDUFS1, NDUFS1-K621R, and control mice in response to Ang II treatment; **H**, AF incidence (left) and quantification of AF duration (right) in control, NDUFS1, and NDUFS1-K621R mice treated with Ang II (n=24–25/group). Data are presented as mean ± SEM. Data were analyzed using the chi-square test for **D** and **H (left)**, and one-way ANOVA followed by Tukey's multiple comparisons test for **B**, **F**, and **H (right)**. *P<0.05, **P<0.01. USP10, Ubiquitin specific peptidase 10; AF, Atrial Fibrillation; Ang II, Angiotensin II; DADs, delayed afterdepolarization; ECG, Electrocardiogram.

Supplemental Tables:

Table S1: Detailed baseline characteristics of the patient cohort.

**Characteristic**	**SR group(n=5)**	**AF group (n=5)**
**Age (years)**	58.2 ± 6.5	63.4 ± 7.2
**Male (%)**	3 (60)	3 (60)
**BMI (kg/m^2)**	24.3 ± 2.8	25.6 ± 3.4
**AF duration (months)**	-	18.6 ± 10.2
**AF type**		
**Persistent AF**	-	4 (80%)
**Paroxysmal AF**	-	1 (20%)
**Left atrial diameter (mm)**	34.8 ± 3.2	44.5 ± 5.6
**Left ventricular ejection fraction, %**	61.5 ± 4.7	58.4 ± 6.5

Table S2: Details of the antibodies used for this study.

Antibodies	Dilution	Catalog#	Vendor
Anti-Usp10	WB: 1:1000; IHC: 1:250	8501S	CST
Anti-Gapdh	WB: 1:5000	60004-1-Ig	Proteintech
Anti-NDUFS1	WB: 1:4000	12444-1-AP	Proteintech
Anti-K48 Ub	WB: 1:2000	8081S	CST
Anti-Flag	WB: 1:3000	M185-3L	MBL
Anti-Myc	WB: 1:3000	M047-3	MBL
Anti-HA	WB: 1:3000	51064-2-AP	Proteintech
HRP-conjugated anti-rabbit IgG	WB: 1:5000	7074	CST
HRP-conjugated anti-mouse IgG	WB: 1:5000	7076	CST

IHC: immunohistochemistry; WB: western blotting.

Table S3: The sequences of RT-qPCR primers

Species	Primer	Sequence（5’-3’）
Human	*USP10* Forward	ATTGAGTTTGGTGTCGATGAAGT
Human	*USP10* Reverse	GGAGCCATAGCTTGCTTCTTTAG
Human	*GAPDH* Forward	GGAGCGAGATCCCTCCAAAAT
Human	*GAPDH* Reverse	GGCTGTTGTCATACTTCTCATGG
Mouse	*Usp10* Forward	GGACTCCATGAAGAGATGCTGAG
Mouse	*Usp10* Reverse	CTCGTCCTCTATCAAGTCGCTTC
Mouse	*Gapdh* Forward	AGGTCGGTGTGAACGGATTTG
Mouse	*Gapdh* Reverse	TGTAGACCATGTAGTTGAGGTCA
