# Supplementary material for: USP10 mitigates Ang II–induced atrial remodeling and atrial fibrillation susceptibility by deubiquitinating NDUFS1
Source: JCI Insight. 2026 Aug 24;11(16):e195761. doi: 10.1172/jci.insight.195761 (PMC13502181; doi:10.1172/jci.insight.195761)

Figure 1C

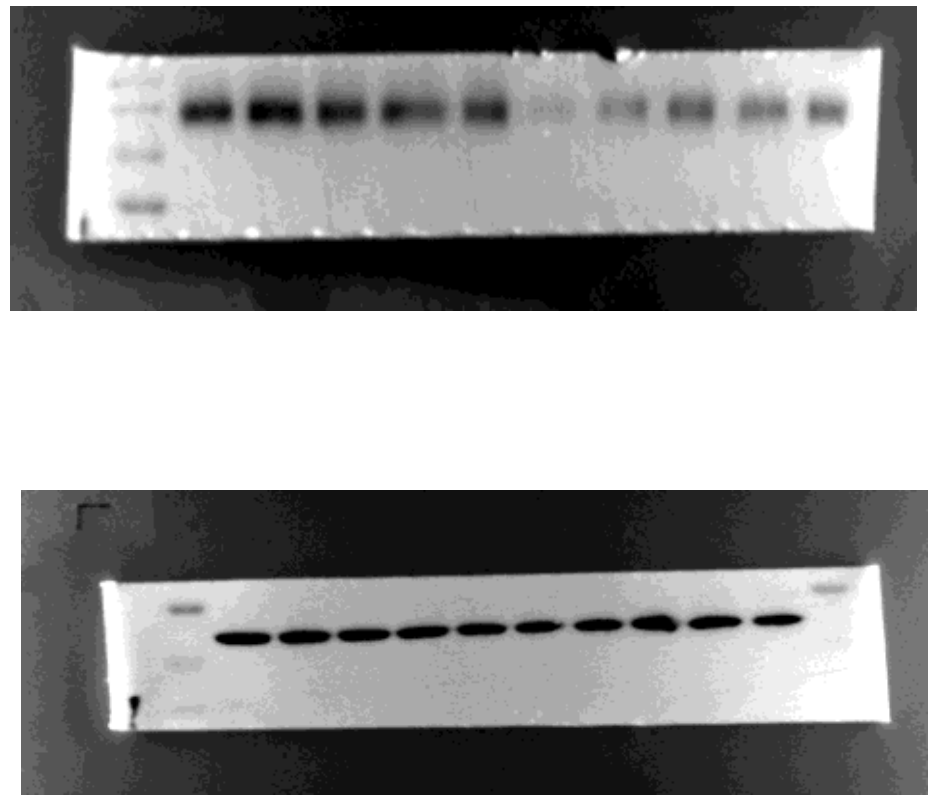

USP10

GAPDH

Figure 1E

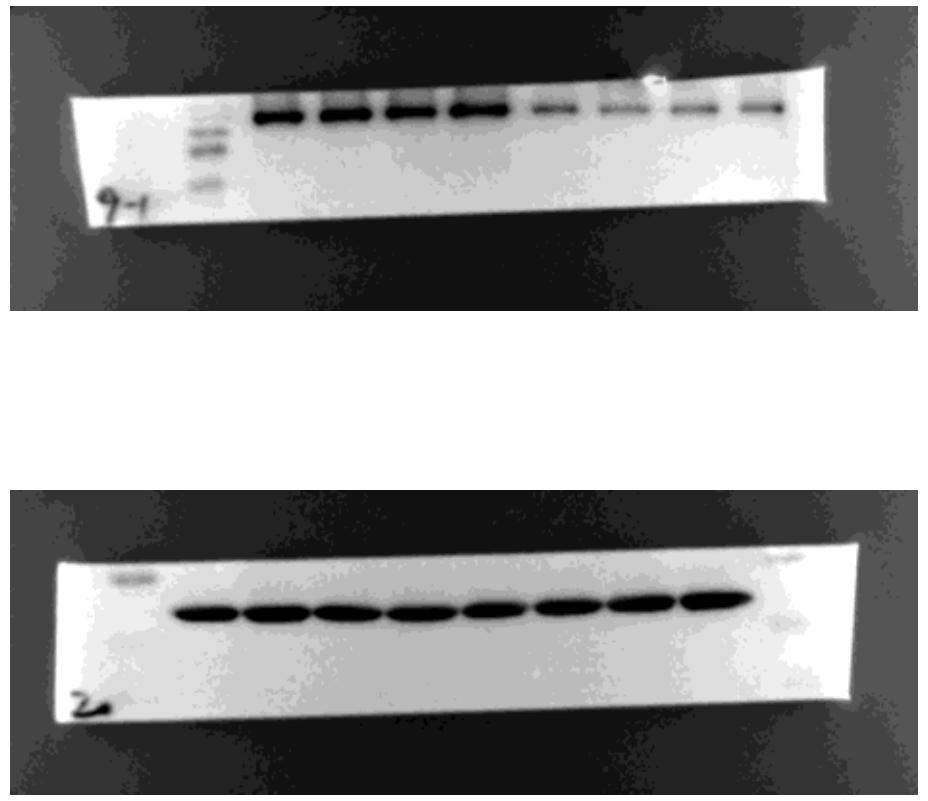

USP10

GAPDH

Figure 1H

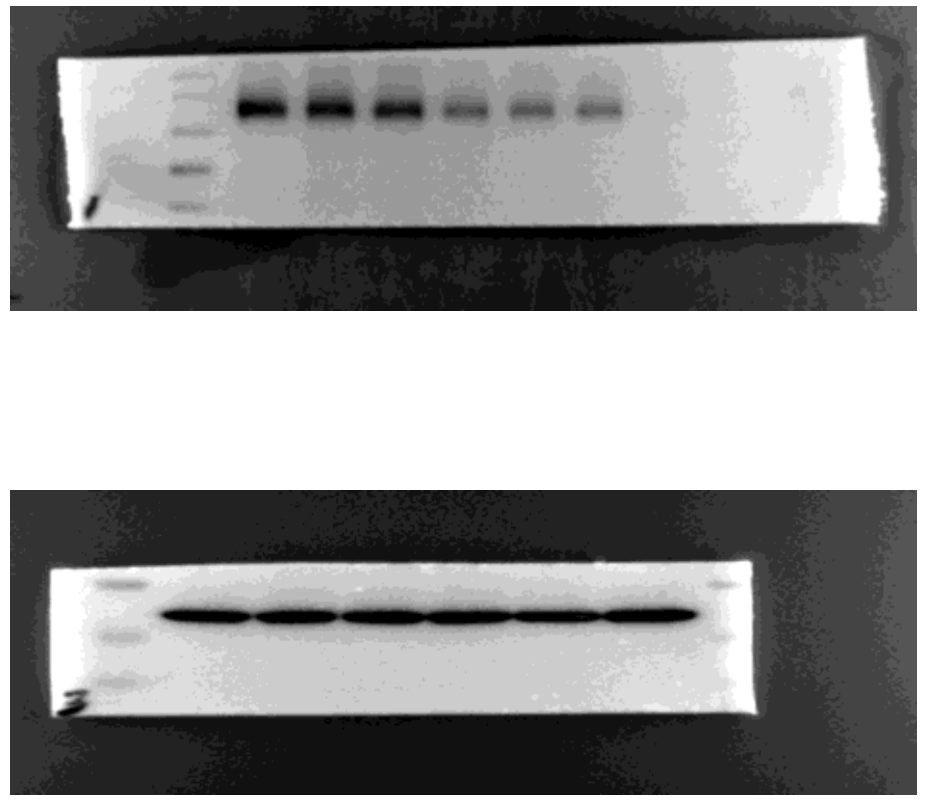

USP10

GAPDH

Figure 5D

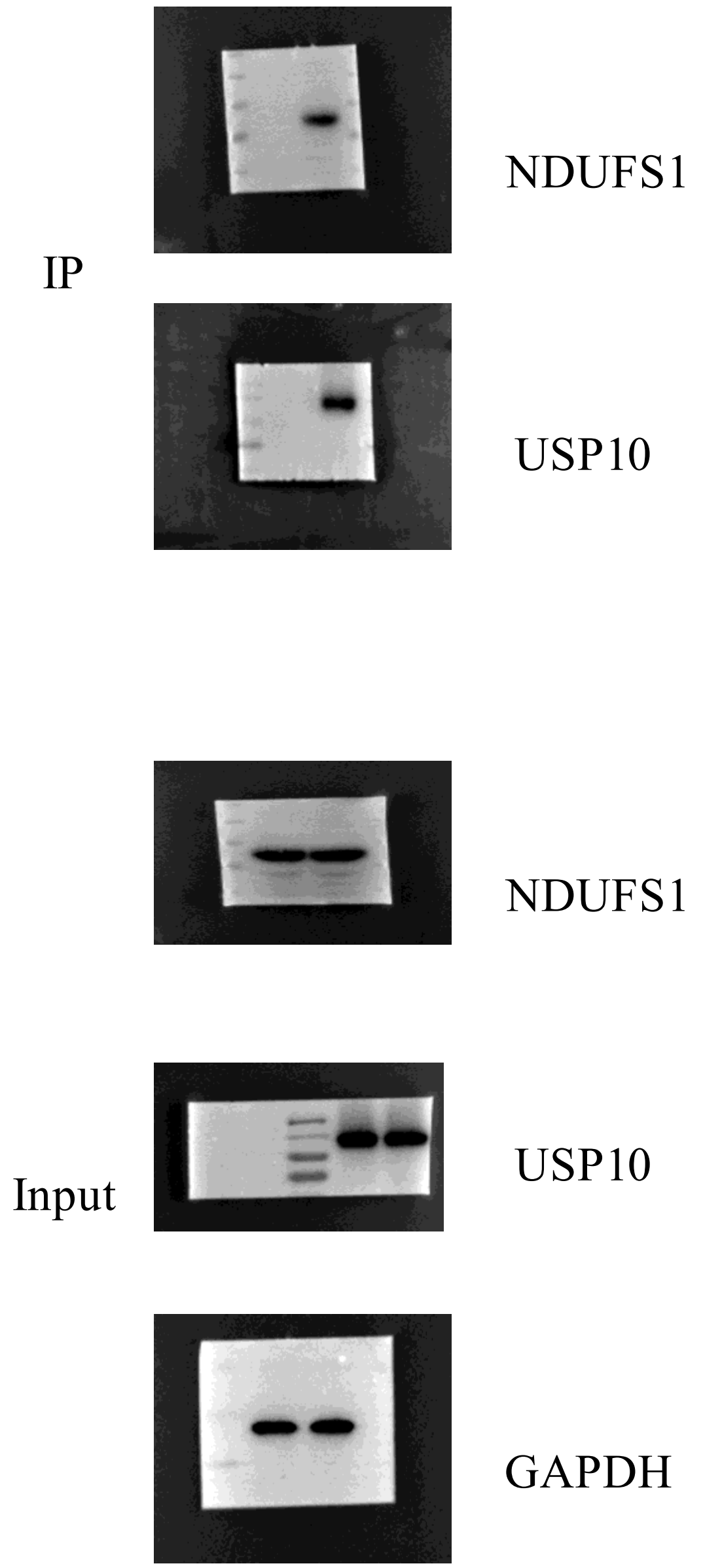

Figure 5E

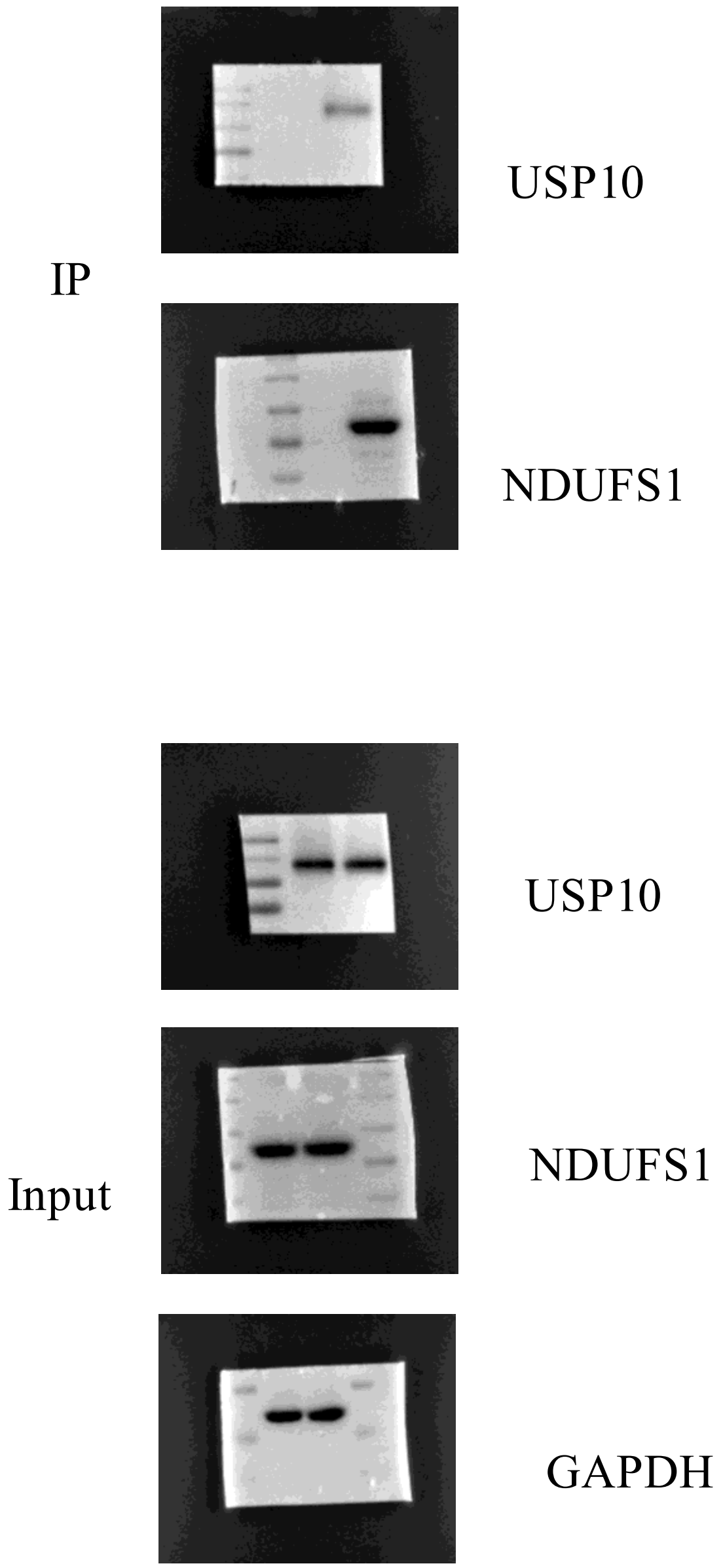

Figure 5F

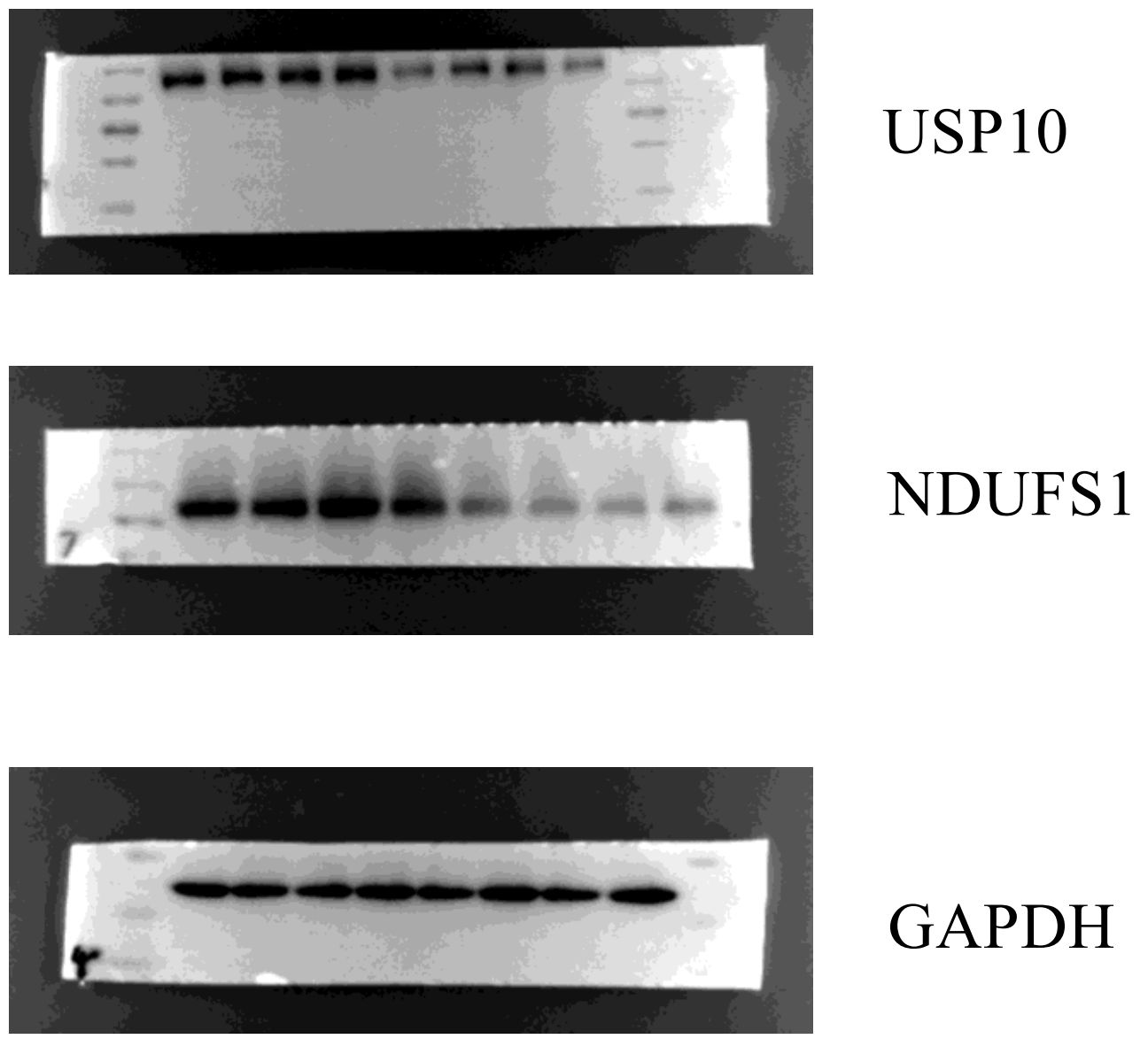

Figure 5G

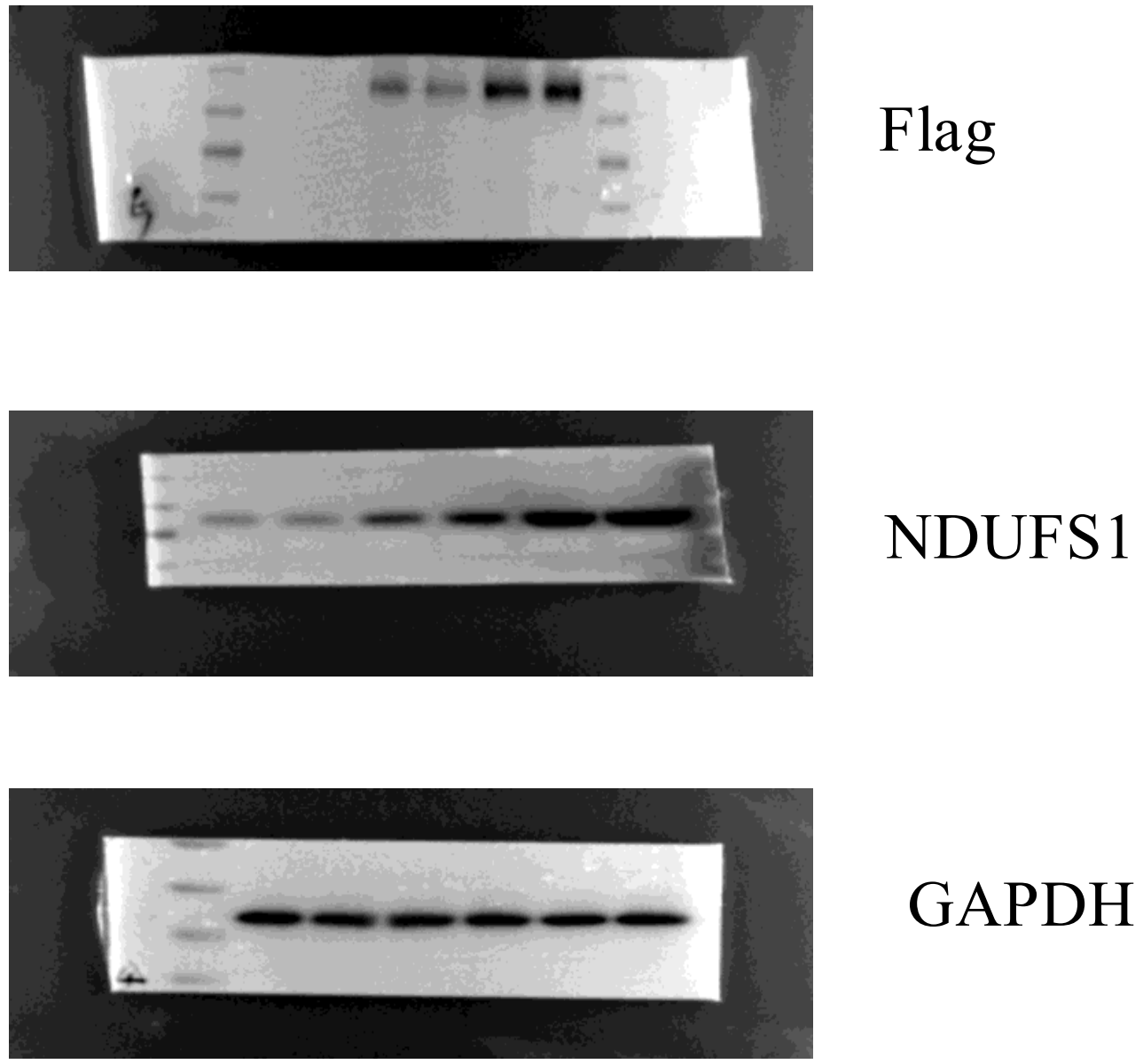

Figure 5G replicate

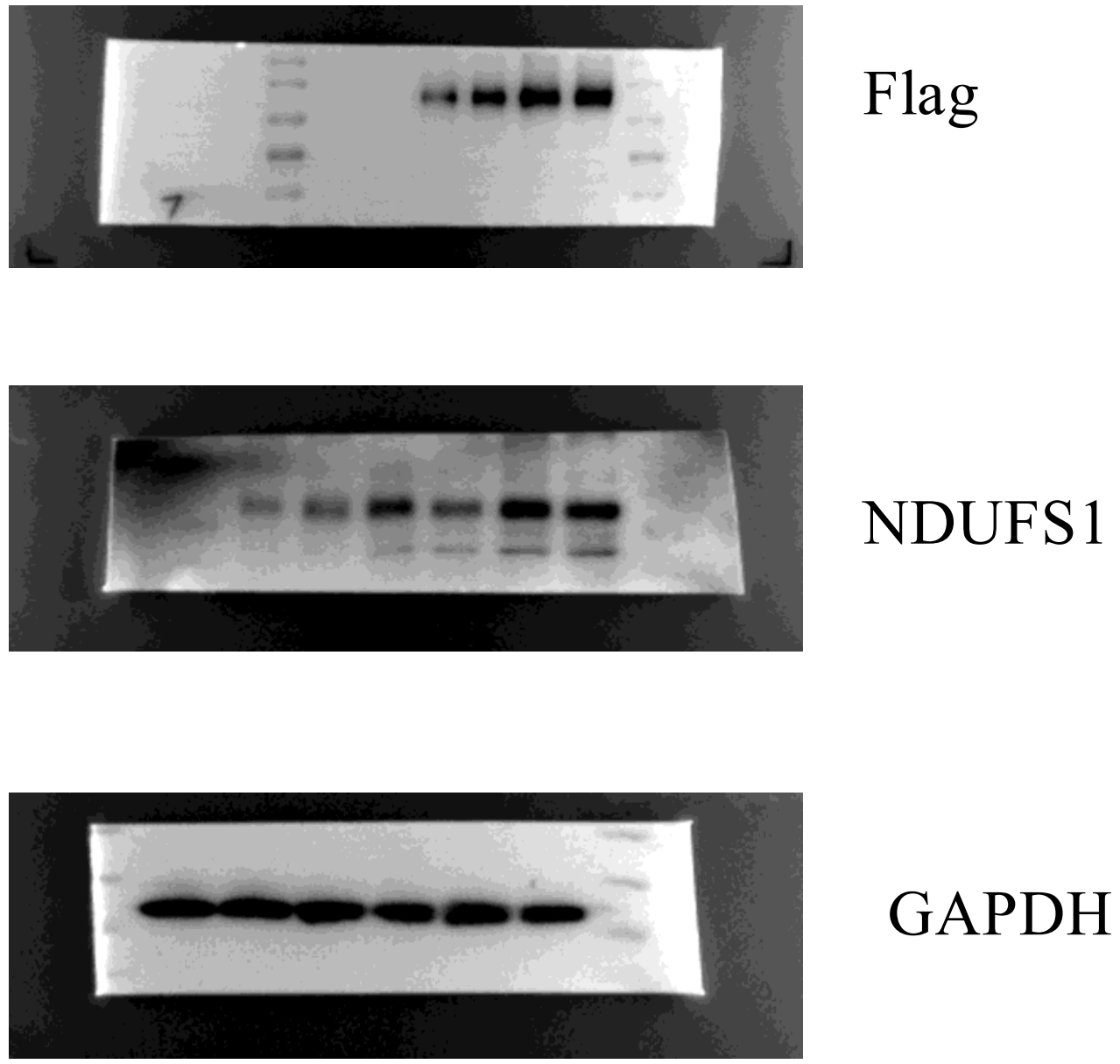

Figure 5H and replicates

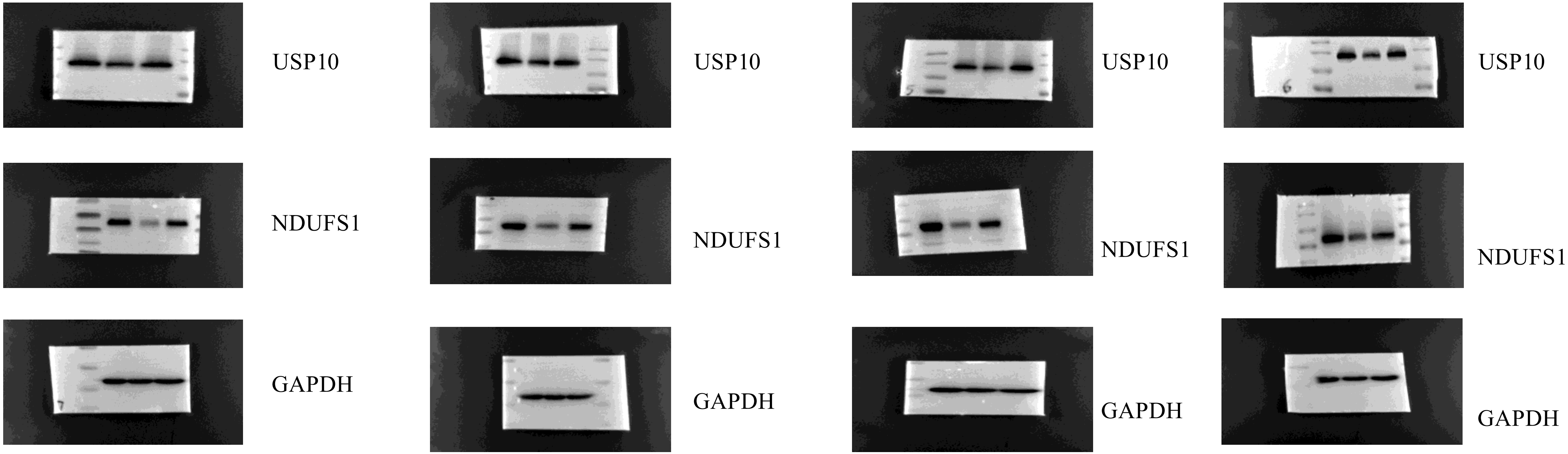

Figure 5I

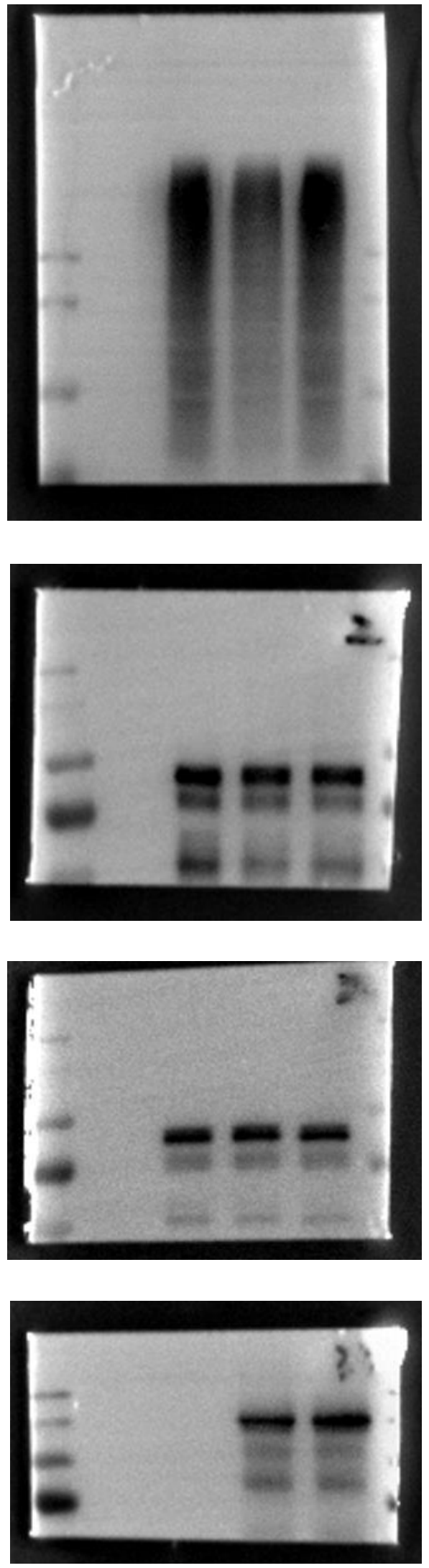

Myc

Flag

Flag

HA

Figure 5J and replicates

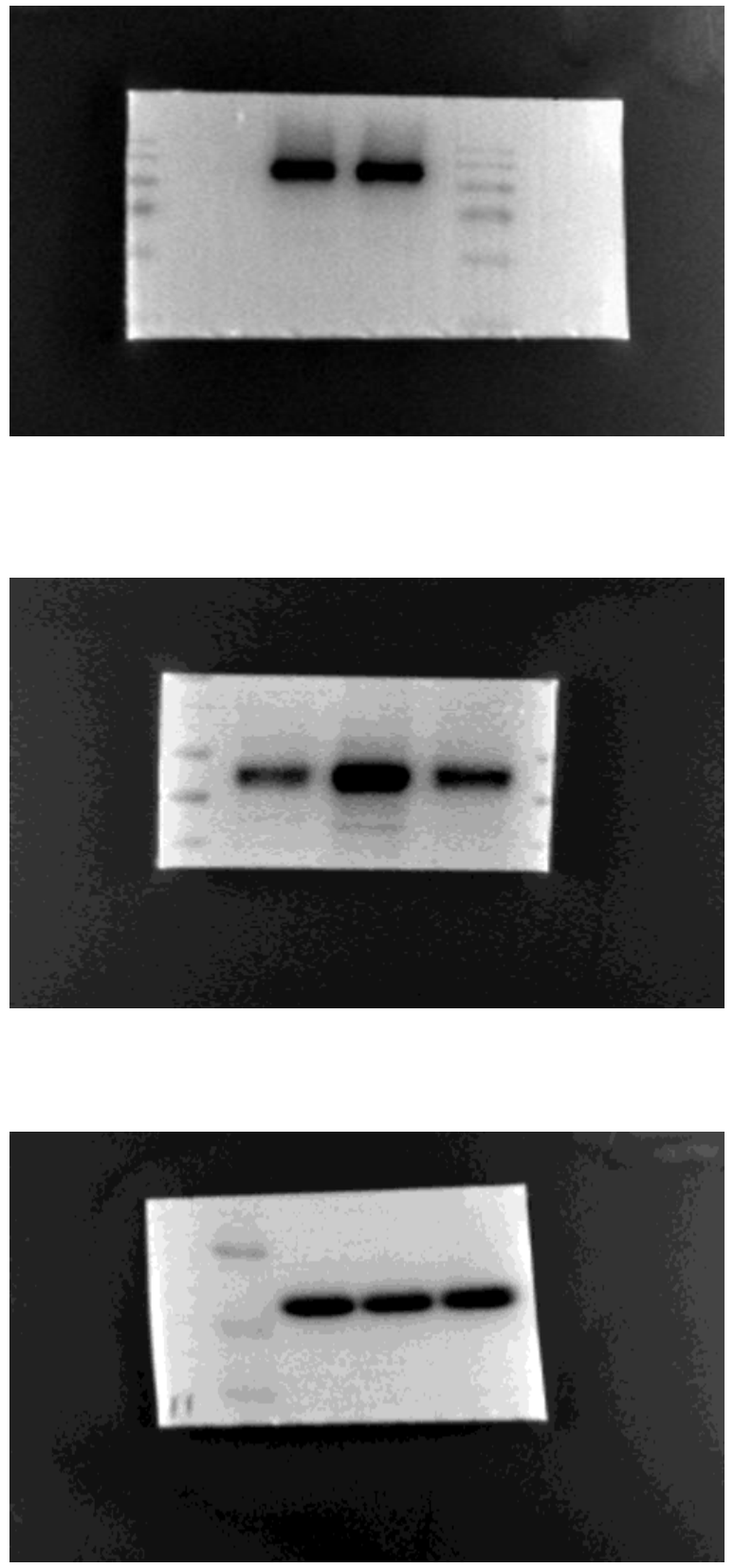

USP10

NDUFS1

GAPDH

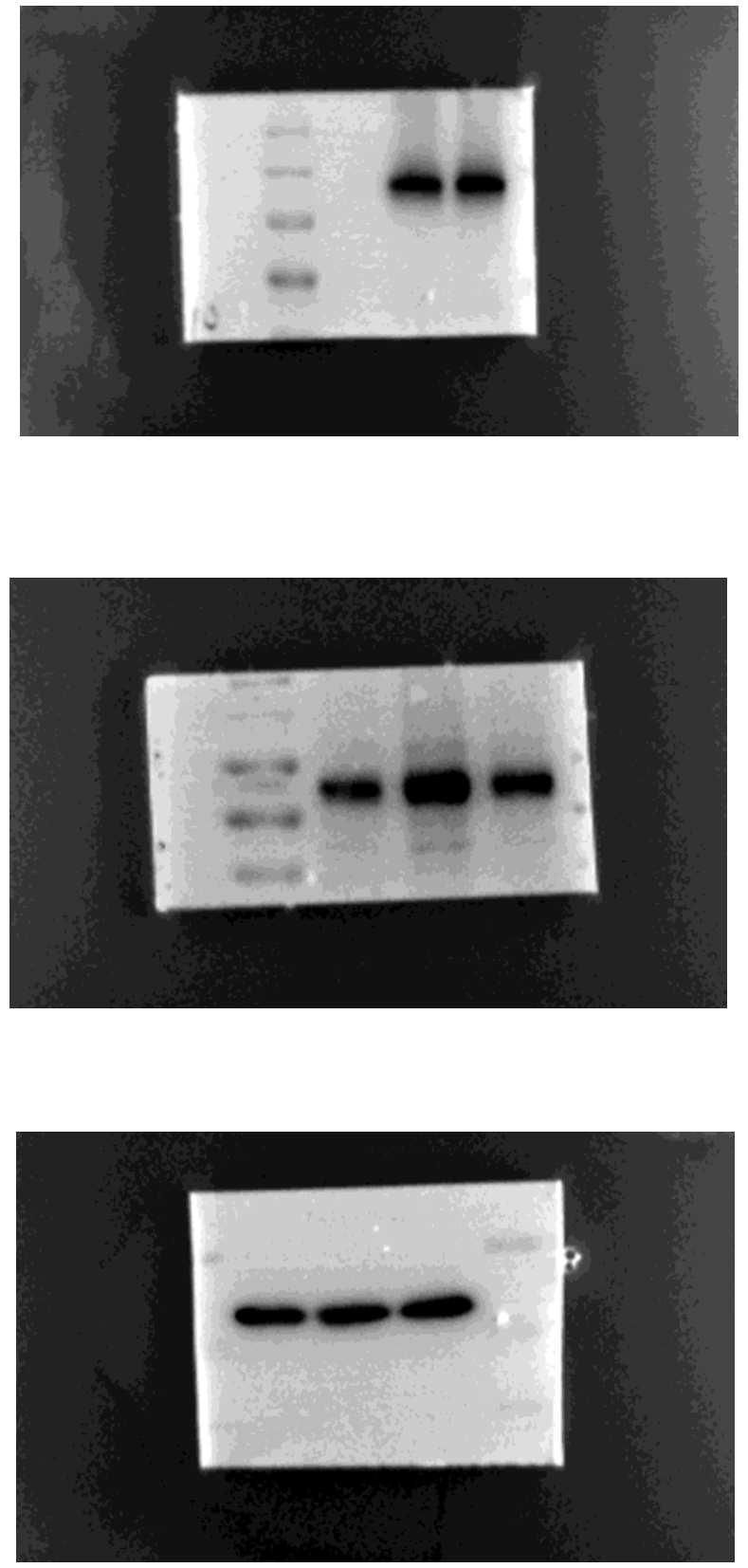

USP10

NDUFS1

GAPDH

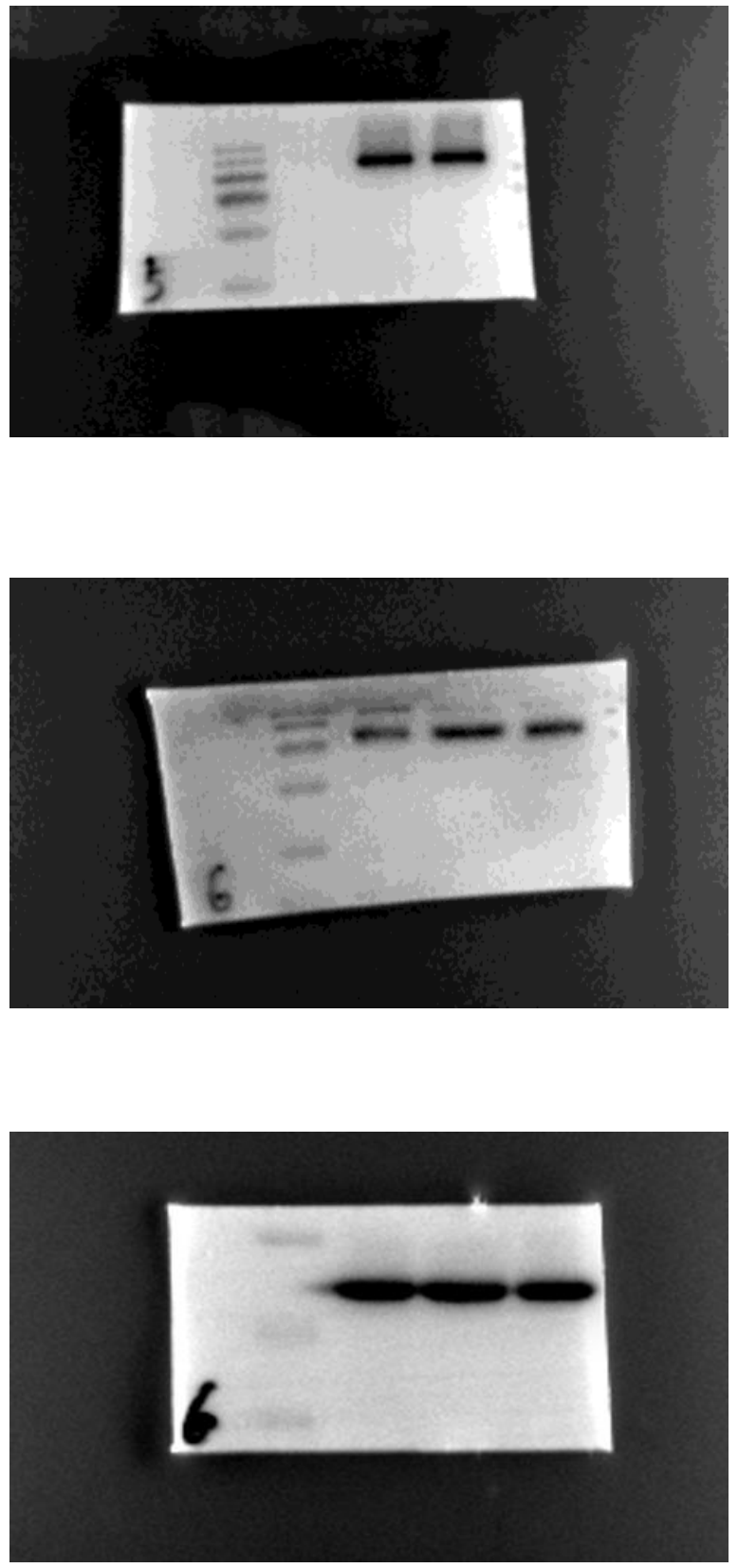

USP10

NDUFS1

GAPDH

Figure 6C

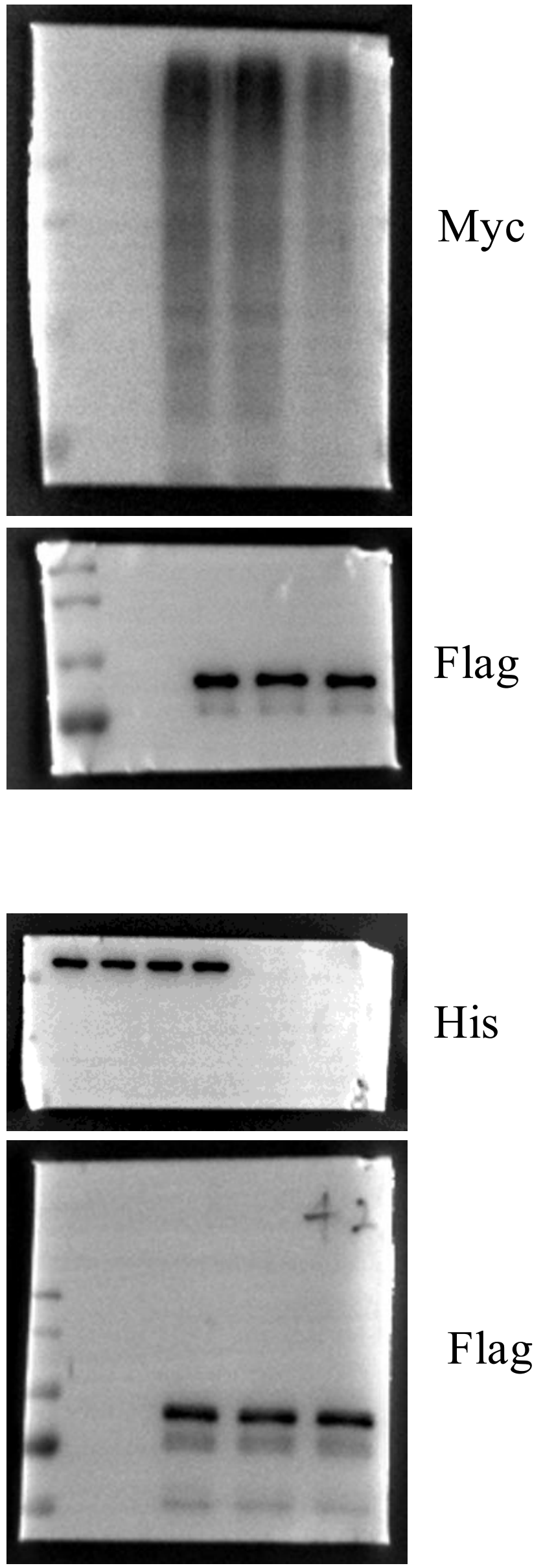

Figure 6D

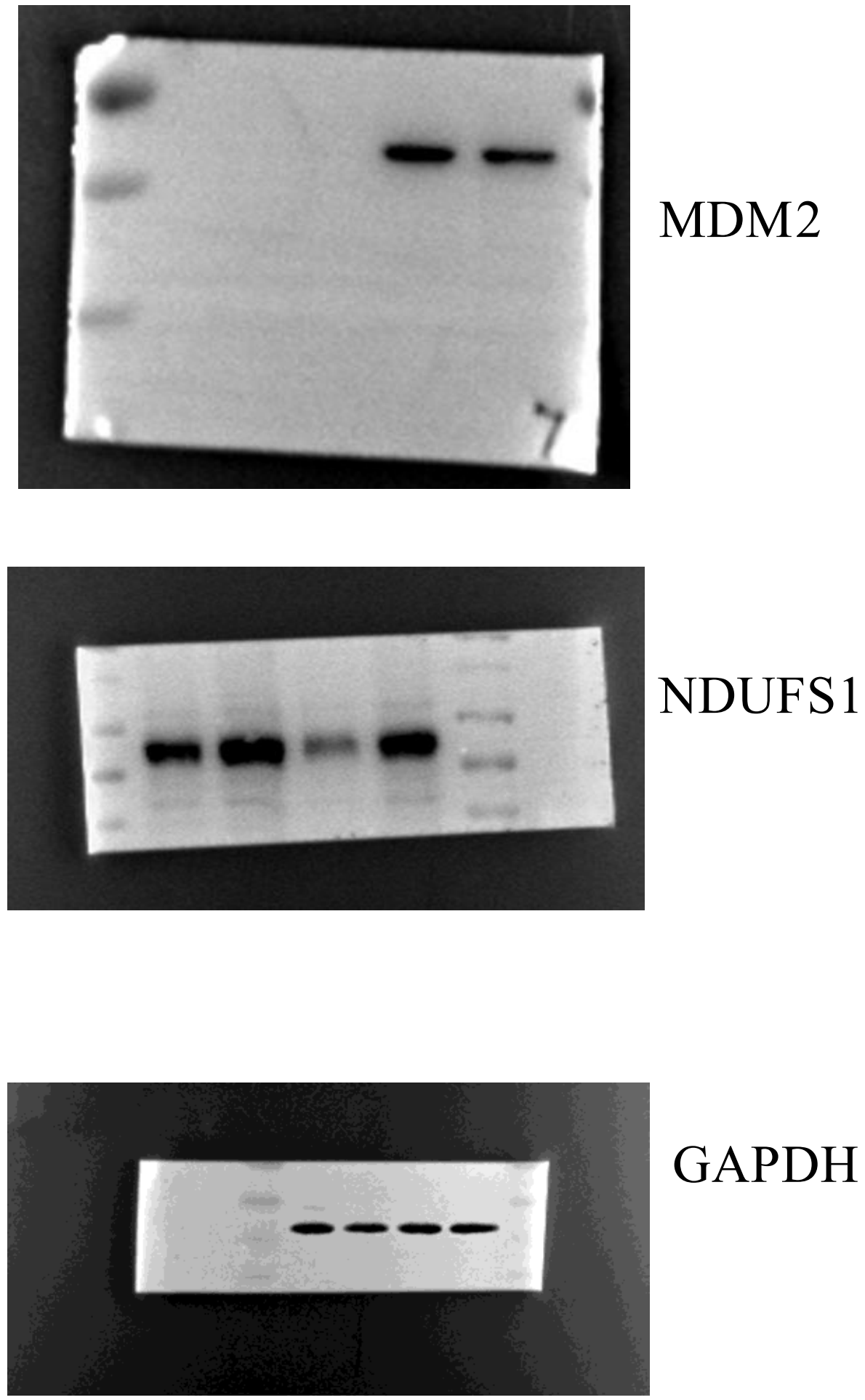

Figure 6F

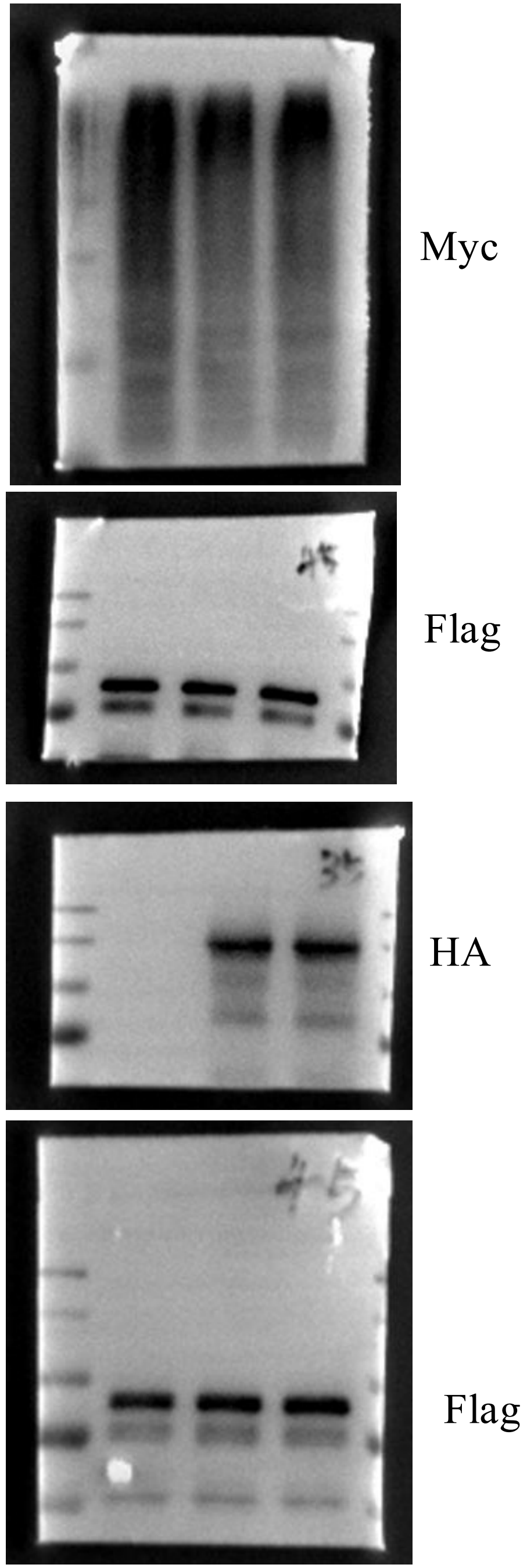

Figure6G

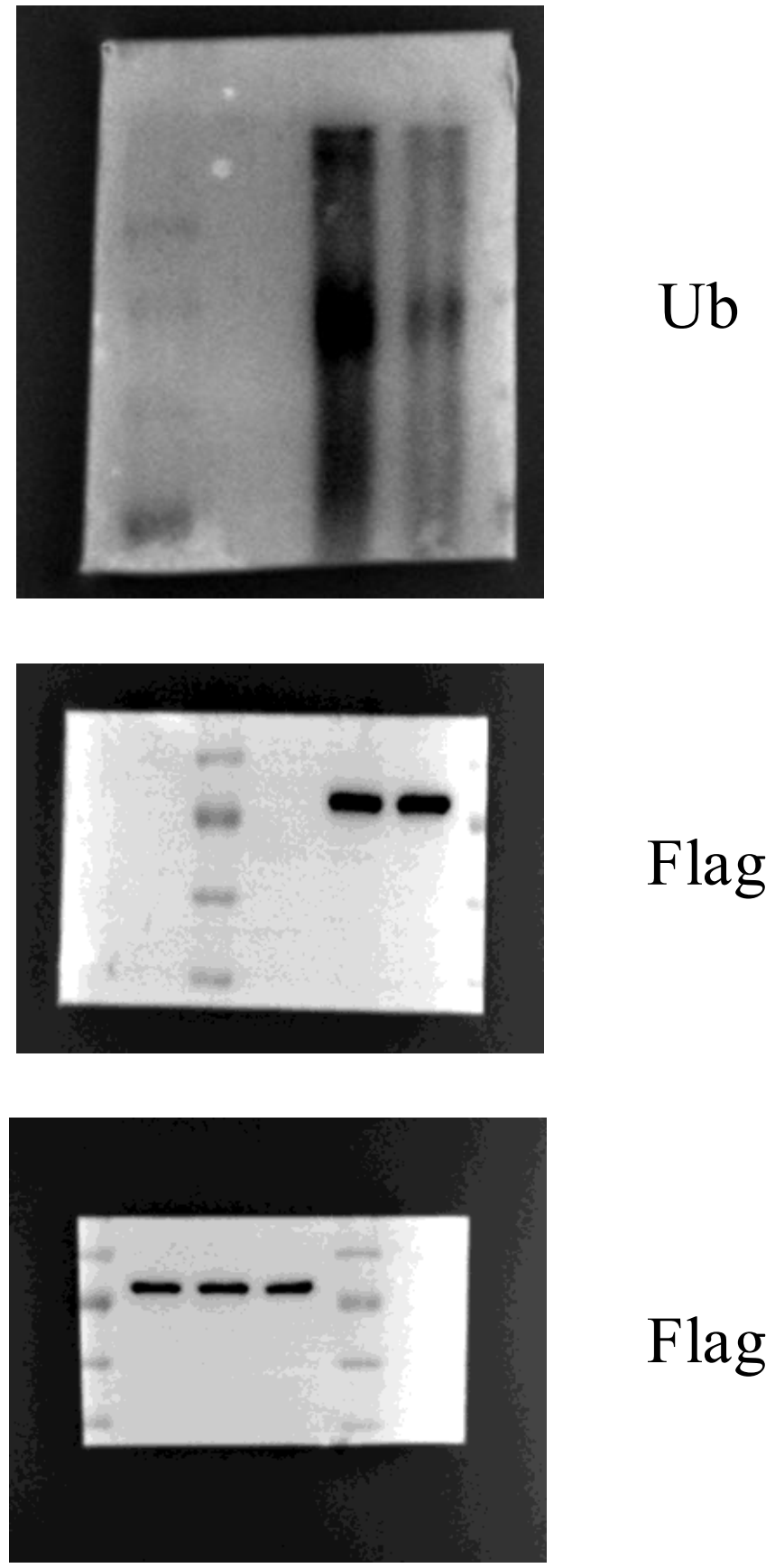

Supplement: Supplementary file 2 [file jciinsight-11-195761-s339.pdf]
